# CMIP6-based global estimates of future aridity index and potential evapotranspiration for 2021-2060

**DOI:** 10.12688/openreseurope.18110.4

**Published:** 2025-05-12

**Authors:** Robert J. Zomer, Jianchu Xu, Donatella Spano, Antonio Trabucco

**Affiliations:** 1 CMCC Foundation -Euro-Mediterranean Center Climate Change, Sassari, Sardinia, Italy; 2 Center for Mountain Futures, Kunming Institute of Botany Chinese Academy of Sciences, Kunming, Yunnan, China; 3 CIFOR-ICRAF China Program, World Agroforestry Centre, Kunming, Yunnan, China; 4 Department of Agricultural Science, University of Sassari, Sassari, Sardinia, Italy; 5 National Biodiversity Future Center - NBFC, Palermo, Sicily, Italy

**Keywords:** Hydrology, Water Cycle, Water Budget, Ecohydrology, Terrestrial Ecosystems, Agroecosystems, Irrigation Management

## Abstract

The “Future_Global_AI_PET Database” provides high-resolution (30 arc-seconds) average annual and monthly global estimates of potential evapotranspiration (PET) and aridity index (AI) for 22 CMIP6 Earth System Models for two future (2021–2041; 2041–2060) and two historical (1960–1990; 1970–2000) time periods, for each of four shared socio-economic pathways (SSP). Three multimodel ensemble averages are also provided (All; Majority Consensus, High Risk) with different level of risks linked to climate model uncertainty. An overview of the methodological approach, geospatial implementation and a technical evaluation of the results is provided. Historical results were compared for technical validation with weather station data ( *PET: r* ^2^ = 0 *.72; AI: r* ^2^ = *0.91*) and the CRU_TS v 4.04 dataset ( *PET: r* ^2^ = 0 *.67;* AI: *r* ^2^ = 0 *.80*). Within the context of projected significant change in the near- and medium-term, the “Future_Global_AI_PET Database” provides a set of data projections and tools available for a variety of scientific and practical applications, illustrating trends and magnitude of predicted climatic and eco-hydrological impacts on terrestrial ecosystems. The Future_Global_AI_PET Database is archived in the ScienceDB repository and available online at: https://doi.org/10.57760/sciencedb.nbsdc.00086

## Introduction

Potential evapotranspiration (PET) characterizes the atmosphere's capacity to remove water through evapotranspiration (ET). Evaporation and transpiration processes collectively transfer water from the Earth's surface to the atmosphere ( Allen *et al.*, 1998; Jensen & Allen, 2016) with rates determined by solar radiation, air temperature, relative humidity (specifically, vapor pressure deficit), wind speed ( Pandey *et al.*, 2017; Zotarelli *et al.*, 2018), as well as the distinctive traits of vegetation, or crops and agricultural practices ( Jensen & Allen, 2016). Estimates of PET have been widely used across diverse scientific fields and practical domains ( Hargreaves, 1994a; Zomer *et al.*, 2022), with PET estimates and related indices playing a crucial role in agricultural and natural resource management with demonstrated utility on scales from individual farms to regional and global ( Anwar *et al.*, 2021; Basarin *et al.*, 2020; Itenfisu *et al.*, 2003; Valipour *et al.*, 2020). While potential evapotranspiration (PET) reflects potential atmospheric water demand across varied vegetation types, useful in broader ecosystem and hydrological studies, reference evapotranspiration (ET _0_) estimates the effective water use from a well-watered reference crop, and as such, is particularly useful for agriculture and irrigation management. 

In an era of rapid environmental and climatic transformations, PET estimates, along with their related indices, assume a pivotal role as direct and critical measures, as well as predictive tools, for gauging the trajectory, direction, and extent of climatic variations and their ramifications for terrestrial, and particularly agricultural and natural ecosystems. As an example, indices relying on PET estimates include the Standardized Precipitation Evapotranspiration Index (SPEI), Palmer Drought Severity Index (PDSI), Crop Water Stress Index (CWSI), Water Deficit Index (WDI), Evaporative Stress Index (ESI), and the Aridity Index (AI) described in this paper, Indices like the SPEI, AI, PDSI, CWSI, WDI, ESI and the AI incorporate PET to understand and evaluate water availability, climate impacts, and drought. Given the current global climate emergency, as reported in recently released findings of the latest Intergovernmental Panel on Climate Change (IPCC) reports ( IPCC, 2019; IPCC, 2021; IPCC, 2022), and other more dire, increasingly urgent assessments ( Forster *et al.*, 2023; Hansen *et al.*, 2023), such estimates and the projection of their trajectory and future trends in the near- and medium-term, not only hold implications for agricultural production, food security, and sustainable development, biodiversity and conservation, but ultimately, the well-being of human civilization.

An established approach employed to evaluate aridity status and changes involves the use of the aridity index (AI), which describes the ratio of precipitation to PET. Aridity indices ( Arora, 2002; Greve *et al.*, 2019; Nastos *et al*., 2013) furnish a means to gauge moisture availability for plant growth, generally of specific reference crops or specific vegetation types ( Allen *et al.*, 1998; Itenfisu *et al.*, 2003; Walter *et al.*, 2001). By consolidating the complex concept of aridity into a single numerical value, the utilization of aridity indices enables both spatial and temporal comparisons. The AI serves as proxy for a comprehensive evaluation of a set of hydro-climatological and hydro-ecological variables to determine historical, recent conditions and projected changes in hydrological cycles at specified future times and time scales, notably soil moisture availability. Application of an AI to historical climate datasets can establish crucial baselines for monitoring and assessing the impact and consequences of climatic change on hydrological cycles. Both PET and AI regularly serve as inputs for various operational decision-making processes, including irrigation management, crop cultivation practices, and the anticipation of drought and flood, with great value for agricultural production and water resource governance ( Zhou *et al.*, 2020). These indices provide policy guidance across various sectors beyond agricultural and natural resource management, with significant implications for meeting the various Sustainable Development Goals, several of which are directly water related.

A previous initial version, the “Global Aridity Index and PET Database” ( Global-AI_PET_v1) ( Trabucco & Zomer, 2008), based on the historical global climatic dataset WorldClim v1.4 ( Hijmans *et al.*, 2005: available at http://WorldClim.org), and calculated using the Hargreaves equation ( Hargreaves, 1994b) for the averaged time period 1960–1990, has been available online since 2009 ( Trabucco & Zomer, 2008; Trabucco *et al.*, 2008; Zomer *et al.*, 2006; Zomer *et al.*, 2008). A subsequent version, the “Global Aridity Index and Potential Evapotranspiration (ET _0_) Climate Database” - Global-AI_PET_v2 ( Trabucco & Zomer, 2019) - implementing a Penman-Monteith equation and based on the WorldClim 2.0 ( Hijmans *et al.*, 2005) for the averaged period 1970–2000, has been available online since 2019, with an updated version - Global-AI_PET_v3 ( Zomer *et al.*, 2022) - based on the WorldClim v2.1 historical dataset available since 2022. These datasets have been shown to have wide utility across a wide range of disciplines and have been downloaded and cited numerous times, and are fully discussed in Zomer *et al.*, 2022. Both have been used in this study as comparative references.

**Figure 1.**
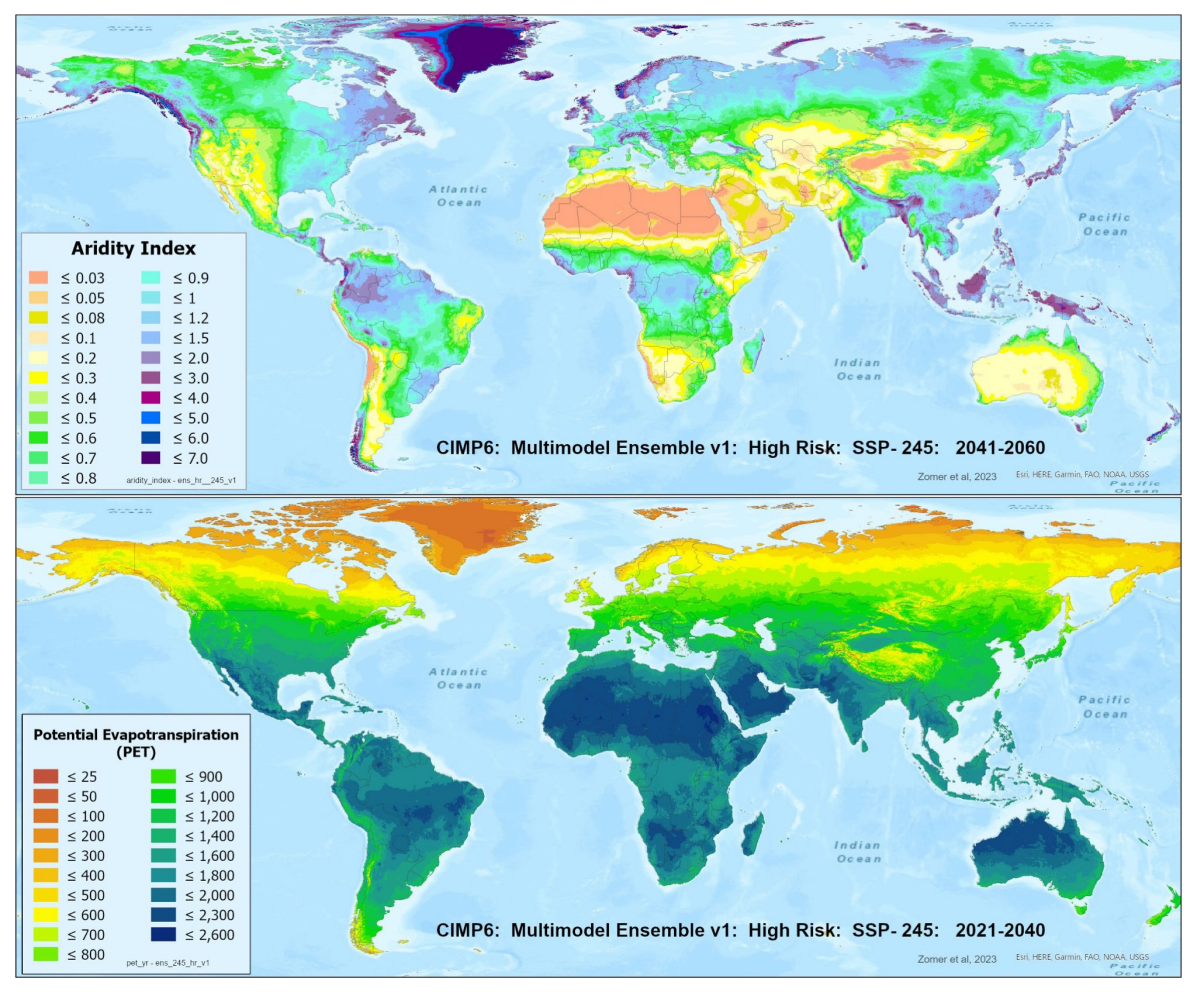
Global PET and Aridity Index. Global PET and Aridity Index calculated using the Hargreaves equation for the entire globe at 1 km spatial resolution, showing an example using a averaged multimodel ensemble of future projections of CMIP6 ESM for two time periods.

A set of future projections of global PET and AI ( Figure 1) were produced to construct the Global-Future_AI_PET Database ( Zomer & Trabucco, 2024) based on 22 Coupled Inter-Comparison Modeling Project – Phase 6 (CMIP6: ( Eyring *et al.*, 2016)) Earth System Models (ESM). Due to a continuing lack of high resolution downscaled CMIP6 future projection data for several climate variables needed to parameterize a full implementation of the Penman-Monteith equation, and a desire to maintain continuity with the previously used WorldClim ( Fick & Hijmans, 2017) datasets (reportedly among the most widely used climatic datasets for environmental, biogeographical or ecological analysis ( Bobrowski *et al.*, 2021)), Hargreaves equation for calculating PET, was applied to the CMIP6 ESM data. This maintains a similar approach and methodology as used to develop the Global-AI_PET Database v1 ( Trabucco & Zomer, 2008) Using bias-corrected downscaled climatic parameters (i.e., minimum / maximum temperature, and precipitation), derived from interpolation of weather station data (30 arc seconds resolution), and provided by WorldClim.org, each ESM model projection was evaluated across four emission scenarios, or *Shared Socio-Economic Pathways* (SSP 126; 245; 370; 585). In total there are 168 ESM/SSP scenario combinations in the database, as there are a few exceptions due to missing data ( Table 1). Additionally, three multimodel ensemble averages have been calculated (for each of the respective SSP): an ensemble including all the available models, a majority consensus ensemble and an ensemble outlining a high risk (more extreme) scenario. In addition, PET and AI have been calculated as 30-years average for two historical time periods (1960–1990; 1970–2000). The Future_Global_AI_PET Database ( Zomer & Trabucco, 2024) described in this paper is archived and available online from the Science Data Bank (ScienceDB) at: https://doi.org/10.57760/sciencedb.nbsdc.00086.

**Table 1.** Available datasets in the *Future_Global_AI_PET Database*. Available datasets in the *Future_Global_AI_PET Database*, including two historical datasets, 22 ESM models calculated for each of their respective SSP, for two time periods, and 3 averaged multimodel ensembles.

Historical Datasets											
WorldClim 1.4 (1960–1990)									ESMs included within Ensemble		
WorldClim 2.1 (1970–2000)											
Earth System Models (ESM)	Year: 2021–2040				Year: 2041–2060				MultiModel Ensemble		
SSP Scenario:	126	245	370	585	126	245	370	585	All	Consensus	High-Risk
ACCESS-CM2	X	X	X	X	X	X	X	X	X		X
ACCESS-ESM1-5	X	X	X	X	X	X	X	X	X	X	
CanESM5	X	X	X	X	X	X	X	X	X		X
CanESM5-CanOE	X	X	X	X	X	X	X	X	X		X
CMCC-ESM2	X	X	X	X	X	X	X	X	X	X	
CNRM-CM6-1	X	X	X	X	X	X	X	X	X	X	
CNRM-CM6-1-HR	X	X	X	X	X	X	X	X	X	X	
CNRM-ESM2-1	X	X	X	X	X	X	X	X	X	X	
FIO-ESM-2-0	X	X		X	X	X		X	X		
GFDL-ESM4	X		X		X		X		X		
GISS-E2-1-G	X	X	X	X	X	X	X	X	X	X	
GISS-E2-1-H	X	X	X	X	X	X	X	X	X	X	
HadGEM3-GC31-LL	X	X		X	X	X		X	X		X
INM-CM4-8	X	X	X	X	X	X	X	X	X	X	
INM-CM5-0	X	X	X	X	X	X	X	X	X	X	
IPSL-CM6A-LR	X	X	X	X	X	X	X	X	X	X	
MIROC-ES2L	X	X	X	X	X	X	X	X	X	X	
MIROC6	X	X	X	X	X	X	X	X	X	X	
MPI-ESM1-2-HR	X	X	X	X	X	X	X	X	X	X	
MPI-ESM1-2-LR	X	X	X	X	X	X	X	X	X	X	
MRI-ESM2-0	X	X	X	X	X	X	X	X	X	X	
UKESM1-0-LL	X	X	X	X	X	X	X	X	X		X
**MultiModlel_Ensemble**									**No. of ESM in Ensemble**		
All	X	X	X	X	X	X	X	X			
Consensus	X	X	X	X	X	X	X	X	20 – 22	15	4 – 5
High-Risk	X	X	X	X	X	X	X	X			

## Methods

### Estimating potential evapotranspiration

Among the various equations employed for estimating PET ( Droogers & Allen, 2002; Hadria *et al.*, 2021; Hargreaves, 1994a; Hargreaves & Samani, 1985; Itenfisu *et al.*, 2003; McMahon *et al*., 2016; Srdić *et al.*, 2023; Sutapa *et al.*, 2020), the Penman-Monteith equation and methodology outlined in the Food and Agriculture Organization FAO-56 ( Allen *et al.*, 1998) guidelines is widely recognized as the standard method. FAO-56 defines and provides guidance on calculating reference evapotranspiration (ET _0_), that is, the evapotranspiration (ET) of a reference crop, rather than defining potential evapotranspiration (PET) directly. FAO-56 presents a standard method to estimate ET _0_, which is widely accepted as a reference standard, and often closely related to PET in practice. In FAO-56, the ET _0_ is calculated using the Penman-Monteith equation, which takes into account factors like temperature, relative humidity, wind speed, and solar radiation. While ET _0_ is a measure of the evapotranspiration rate from a reference crop under optimal conditions, PET generally represents the water demand of vegetation if water is freely available. Though not synonymous, ET _0_ from FAO-56 is widely used as an estimate for PET, particularly in agricultural and environmental applications. 

FAO-56 defined the ET of a reference crop (ET _0_) under optimal conditions. Specifically, it assumes a reference crop characterized by well-watered grass with a uniform height of 12 centimeters, a fixed surface resistance of 70 seconds per meter, and an albedo of 0.231. More generally, ET _0_ quantifies the rate at which readily available soil water evaporates from specified vegetated surfaces comprised of dense, actively growing vegetation with prescribed height and surface resistance, not limited by soil water availability and encompassing an expanse of at least 100 sq. meters of similar or identical vegetation types. ET _0_ serves as a fundamental hydrological variable with utility in various research domains, notably related to hydrologic water balance, simulation of crop yield, management of irrigation systems, and water resources management. ET _0_ values obtained from different locations or seasons are comparable as they pertain to evapotranspiration from the same reference surface enabling researchers and practitioners to evaluate atmospheric evaporative demand independent of specific crop characteristics, crop development stages, management practices, or water availability ( Itenfisu *et al.*, 2003; Zotarelli *et al.*, 2018). Climatic parameters and crop-specific resistance coefficients determined for the reference vegetation are the principal factors influencing ET _0_ estimation ( Jensen & Allen, 2016). As such, additional crop-specific coefficients (Kc) and ET _0_ can, in turn, be employed to derive ET estimates for specific crops (ETc).

The International Commission for Irrigation and Drainage (ICID) ( Doorebos & Pruitt, 1977; Hargreaves, 1994b) and the Food and Agriculture Organization of the United Nations - FAO ( Allen *et al.*, 1998) designated the Penman-Monteith (PM) method as a standard method of computing ET _0_ from climatic data and designated it as the reference for evaluating other methods. A study of ET _0_ equations using lysimeter ET and synoptic climatic data by European Commission’s Joint Research Centre ( Choisnel *et al.*, 1992) compared twelve equations using Penman-Monteith ( Allen *et al.*, 1998) as the reference standard. The results from the PM equation were compared with nine equations that are simpler and require less data than the data intensive requirements of the PM equations. The Hargreaves ( Hargreaves, 1994b) equation demonstrated the nearest results to the classic PM ( Droogers & Allen, 2002; Itenfisu *et al.*, 2003). The Hargreaves method with substantially less data requirements, i.e., requiring only measured values of maximum and minimum temperatures and precipitation, was recommended for more general use and therefore is considered more appropriately applicable to data scarce regions and situations ( Droogers & Allen, 2002). For the purposes of this paper, to maintain clarity, we refer to reference evapotranspiration calculated using the Penman-Monteith as “ET _0_” and calculations using the Hargreaves equation as “PET”.

**Figure 2.**
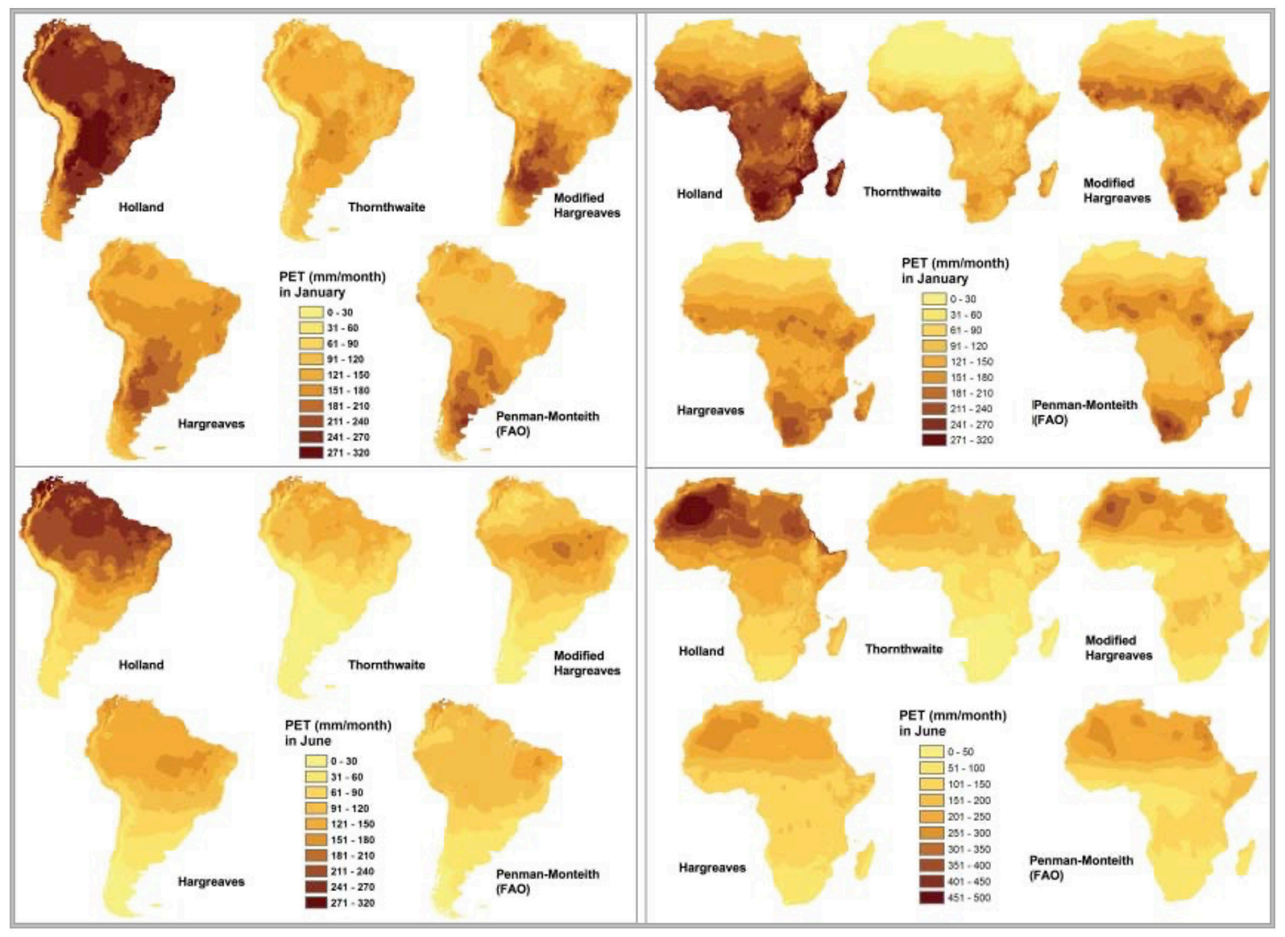
Comparison of five different methods of calculating PET. Comparison of four different temperature-based methods of calculating PET (Thornthwaite, Thornthwaite modified by Holland, Hargreaves, Hargreaves Modified by Droogers and Allen), against the reference FAO Global Penman–Monteith.

To meet the objectives of a global spatial analysis at the desired very high spatial resolution (30 arc seconds or ~1 km) with available climate variables (temperature and precipitation), in the preparation of the previously released first version of the *Global-AI_PET_v1* ( Trabucco *et al.*, 2008; Zomer *et al.*, 2006), four different temperature-based methods of calculating PET were compared with the Penman-Monteith method for calculating ET _0_, and tested to determine which equation performed the best for the objectives of that analysis ( Figure 2; Table 2): Thornthwaite ( Thornthwaite, 1948), Thornthwaite modified by Holland ( Holland, 1978), Hargreaves ( Hargreaves, 1994a), Hargreaves Modified by Droogers and Allen ( Droogers & Allen, 2002), and the FAO Global Penman–Monteith Spatial Dataset ( Allen *et al.*, 1998).

**Table 2.** Comparison of four different temperature-based methods of calculating PET ( Zomer *et al.*, 2006). Five different methods of calculating PET were tested to verify which performed the best for the objectives of this analysis: Thornthwaite ( Thornthwaite, 1948), Thornthwaite modified by Holland ( Holland, 1978), Hargreaves ( Hargreaves *et al.*, 1985), Hargreaves modified by Droogers ( Droogers & Allen, 2002), and the FAO Global Penman-Monteith Dataset ( Allen *et al.*, 1998). Results of the comparison are given as the mean difference (Mean Diff) between Penman-Monteith estimates based on observed weather station data and predicted estimates using the various different methods, and their standard deviations (Std Dev). Source: ( Trabucco *et al.*, 2008).

		Mean Difference (mm) and Standard Deviation (mm) between Observed and Predicted Values:									
		Holland (Thornthwaite)		Thornthwaite		Hargreaves		Modified Hargreaves		Penman-Monteith FAO	
Region	Month	Mean Diff	*Std Dev*	Mean Diff	*Std Dev*	Mean Diff	*Std Dev*	Mean Diff	*Std Dev*	Mean Diff	*Std Dev*
**Africa**											
	January	71.8	*40.2*	41.6	*33.3*	22.3	*16.1*	24.8	*20.1*	11.1	*12.6*
	July	84.4	*41.7*	32.1	*23.7*	20.0	*19.3*	21.1	*19.3*	12.7	*16.0*
**South America**											
	January	69.9	*43.6*	50.5	*32.9*	38.2	*19.2*	41.6	*26.0*	34.9	*26.7*
	July	67.3	*35.9*	37.2	*24.7*	27.2	*14*	30.4	*20.1*	24.3	*15.1*
**Resolution**		1 km		1 km		1 km		1 km		20 km	

These are briefly described below:

**Thornthwaite equation** ( Thornthwaite, 1948)


$$PET=16\left(\frac{L}{12}\right)\left(\frac{N}{30}\right){\left(\frac{10T_{d}}{I}\right)}^{\propto }\;(1)$$

Where:

*PET* is the estimated potential evapotranspiration (mm/month)

*T _d_* is the average daily temperature (degrees Celsius; if this is negative, use 0) of the month being calculated

*N* is the number of days in the month being calculated

*L* is the average day length (hours) of the month being calculated

∝ = (6.75 * 10 ^–7^) *I* ^3^ – (7.71 * 10 ^–5^) *I* ^2^ + (1.792 * 10 ^–2^) *I* + 0.49239 

$I={\sum_{i=1}^{12}\left(\frac{T_{m_{i}}}{5}\right)}^{1.54}$ is a heat index based on the 12 monthly mean temperatures. 

**Thornthwaite modified by Holland** ( Gordon *et al.*, 2005; Holland, 1978):

This equation requires only one climatic input variable (Celsius temperature *T*) and it has been suggested that Thornthwaite-based methods are as good as more refined methods when dealing with monthly data as compared with more detailed daily data ( 27).


$$PET=1.2*10^{9}*e[-4.62*10^{3}/(T+273.15)]\;(2)$$

**Modified Hargreaves** ( Droogers & Allen, 2002)

ET _0_ = 0.0013 · 0.408RA · (Tavg + 17.0) · (T D − 0.0123P)0.76           (3)

Where:

*RA* is extraterrestrial radiation expressed in (MJ m−2 d−1),

*T*avg is average daily temperature (C°)

*TD* (C°) is the temperature range

*P* is precipitation in mm per month

**FAO 56 Penman–Monteith equation** ( Allen *et al.*, 1998)

The FAO-56 Penman-Monteith form of the combination equation to estimate ET _0_ is calculated as:


$$ETo=\frac{\Delta (R_{n}-G)+\rho_{a}c_{p}\frac{\left(e_{s}-e_{a}\right)}{r_{a}}}{\Delta +\gamma (1+\frac{r_{s}}{r_{a}})}\;(4)$$

Where

*ET _0_* is the evapotranspiration for reference crop, as mm day ^-1^

*R _n_* is the net radiation at the crop surface, as MJ m ^-2^ day ^-1^

*G* is the soil heat flux density, as MJ m ^-2^ day ^-1^

*c _p_* is the specific heat of dry air

*p _a_* is the air density at constant pressure

*e _s_* is the saturation vapour pressure, as *kPa*

*e _a_* is the actual vapour pressure, as *kPa*

*e _s_* - e _a_ is the saturation vapour pressure deficit, as *kPa*

Δ is the slope vapour pressure curve, as *kPa* °C ^-1^

*γ* is the psychrometric constant, as *kPa* °C ^-1^

*r _s_* is the bulk surface resistance, as m s ^-1^

*r _a_* is the aerodynamic resistance, as m s ^-1^

Values for PET calculated using each of the above five methods were compared to PET values for specific weather stations calculated using historical weather station data (n = 2288). ET _0_ values were calculated using the more complex Penman–Monteith model applied on direct observations of the various climatic parameters obtained from the FAOCLIM ( FAO and (SDRN), 2001) climate station dataset (covering the time period: 1960–1990). Based on the results of the comparative validation, the Hargreaves model was confirmed to be the best fit to model PET globally with the available data at the time and chosen for the implementation of the *Global-AI_PET_v1*. The Hargreaves method has been shown to perform well compared to the PM method, but required less parameterization, and had significantly lower sensitivity to error in climatic inputs ( Allen *et al.*, 1998; Droogers & Allen, 2002; Jensen *et al.*, 1997; Pimentel *et al.*, 2023). Although the release of the *WorldClim v2.1* allowed for the implementation of the PM equation and the development of the *Global-AI_PET_v3*, the currently available downscaled CMIP6 projections (available from *WorldClim*.org) do not provide the full set of climate variables required to implement the PM equation at this resolution.

### Hargreaves equation

The Hargreaves methodology ( Hargreaves, 1994b; Hargreaves & Samani, 1985; Jensen & Allen, 2016; Jensen *et al.*, 1997; Šimůnek *et al.*, 2005) uses only mean monthly temperature ( *T _mean_*), mean monthly temperature range ( *TD*) and extraterrestrial radiation ( *RA*, radiation on top of the atmosphere) to calculate PET, as shown below:

$$PET=0.0023*R_{a}*(T_{mean}+17.8)*TD^{0.5}\;(5)$$

where *R _a_* is extraterrestrial radiation at the top of the atmosphere, TD is the difference between mean maximum temperatures and mean minimum temperatures *(T _max_ - T _min_), and T _mean_ is equal to T _max_* + *T _min_* divided by 2. *PET is* multiplied by number of days in each month to give the monthly total (mm/month).

Extraterrestrial radiation, *R _a_*, is calculated as follows ( Jensen *et al.*, 1997; Jensen & Allen, 2016; Šimůnek *et al.*, 2005):

$$R_{a}=\frac{0.408\;G_{sc}}{\pi }d_{r}(\omega_{s}\sin\varphi \sin\delta +\cos\varphi \cos\delta \sin\omega_{s})\;(6)$$

Where: *R _a_* is in mm/day

*G _sc_* is the solar constant [MJ m ^-2^day ^-1^] (117.5 MJ m ^-2^day ^-1^),

*φ* is the latitude [radians],

*ω _s_* is the sunset hour angle [radians],

*d _r_* is the relative distance between Earth and Sun

*δ* is the solar declination [rad].

The last three variables are calculated as follows:

$$\omega_{s}=\text{arccos}\;(-\tan\varphi \tan\delta )\;(7)$$

$$d_{r}=1+0.033\cos\left(\frac{2\pi }{365}J\right)\;(8)$$

$$\delta =0.409\;\sin\left(\frac{2\pi }{365}J-1.39\right)\;(9)$$

Where:   J is the Julian Day, or number of the day in the year.

The Hargreaves equation was implemented globally on a per grid cell basis at 30 arc seconds resolution (~ 1km2 at the equator), in ArcGIS (v11.1) using Python v3.2 (see code availability section) to estimate PET and derive an AI using an ensemble of future projections provided by the CMIP6 collaboration ( Eyring *et al.*, 2016). The data to parametrize the equation were obtained from the WorldClim.org online data repository ( Fick & Hijmans, 2017), which provides bias-corrected downscaled monthly values of minimum temperature, maximum temperature, and precipitation for 22 CMIP6 Earth System Models (ESMs), for each of four SSP: 126, 245, 370 and 585.

PET and AI were estimated for two historical periods, WorldClim 1.4 (1960–1990) and WorldClim 2.1 (1970–2000), representing on average a decades’ change, by applying the Hargreaves methodology described above. Similarly, PET and AI were estimated for two future time periods, namely 2021–2040 and 2041–2060, for each of the 22 models across their respective four SSP scenarios (126, 245, 370,585).

### Aridity Index

The aridity index (AI) is expressed as the ratio of precipitation over PET (or ET _0_). The AI quantifies water availability for plant growth based on the ratio of precipitation to PET (or ET _0_) and is a measure of available precipitation relative to water demand for plant growth ( Arora, 2002; Derdous *et al.*, 2020; Girvetz & Zganjar, 2014).

The AI for the averaged time periods has been calculated on a per grid cell basis, as:

AI=MA_Prec/MA_PET(10)

where:

*AI* = Aridity Index

*MA_Prec* = Mean Annual Precipitation

*MA_PET* = Mean Annual PET

Using the mean annual precipitation ( *MA_Prec*) values obtained from the CMIP6 downscaled climate projections, and the PET ( *MA_PET*) datasets estimated as monthly averages by the method described above and aggregated at yearly scale. The *AI* algorithm was implemented on a per grid cell basis. Using this formulation, *AI* values are unitless, increasing with more humid condition and decreasing with more arid conditions.

As a general reference, the climate classification scheme for AI values provided by UNEP ( UNEP, 1997) provides an insight into the climatic significance of the range of moisture availability conditions described by the AI ( Table 3). Higher values of the AI, primarily evident in the colder and arctic regions, are considered not interpretable in terms of plant growth as per the original intent of the aridity index measure.

**Table 3.** Climate classification scheme for AI values. Climate classification scheme for AI values ( UNEP, 1997).

Aridity Index Value	Climate Class
< 0.03	Hyper-Arid
0.03 – 0.2	Arid
0.2 – 0.5	Semi-Arid
0.5 – 0.65	Dry Sub-Humid
> 0.65	Humid

### Multi-model ensembles

**Figure 3.**
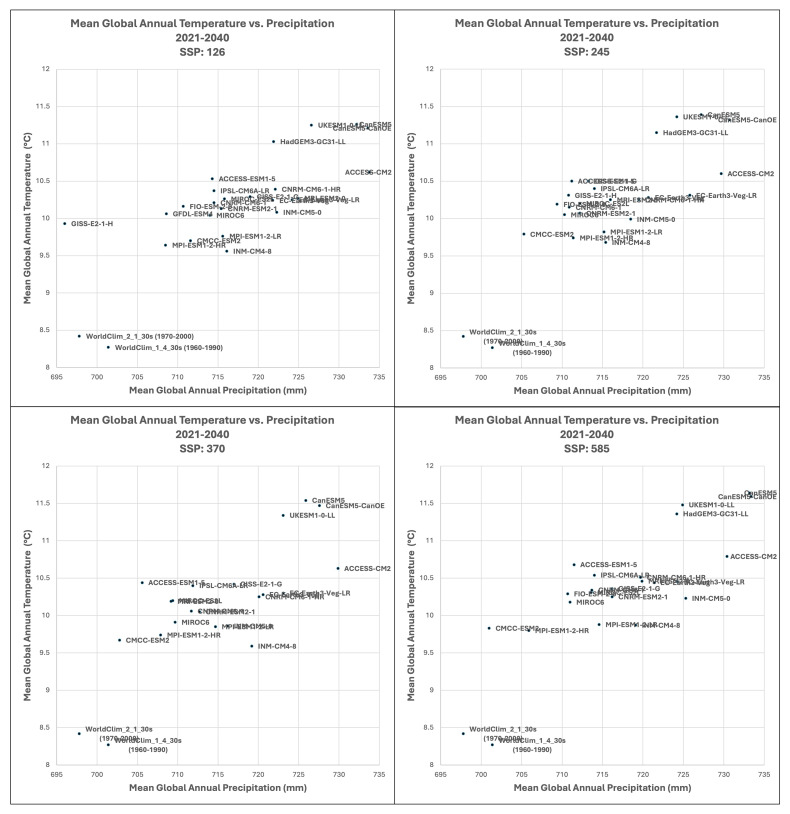
Distribution of 22 CMIP6 Earth System Models along temperature and precipitation gradients (2021–2040). Distribution of average global mean annual temperature and mean annual precipitation of 22 CMIP6 ESM for four SSP for the time period 2021–2040.

**Figure 4.**
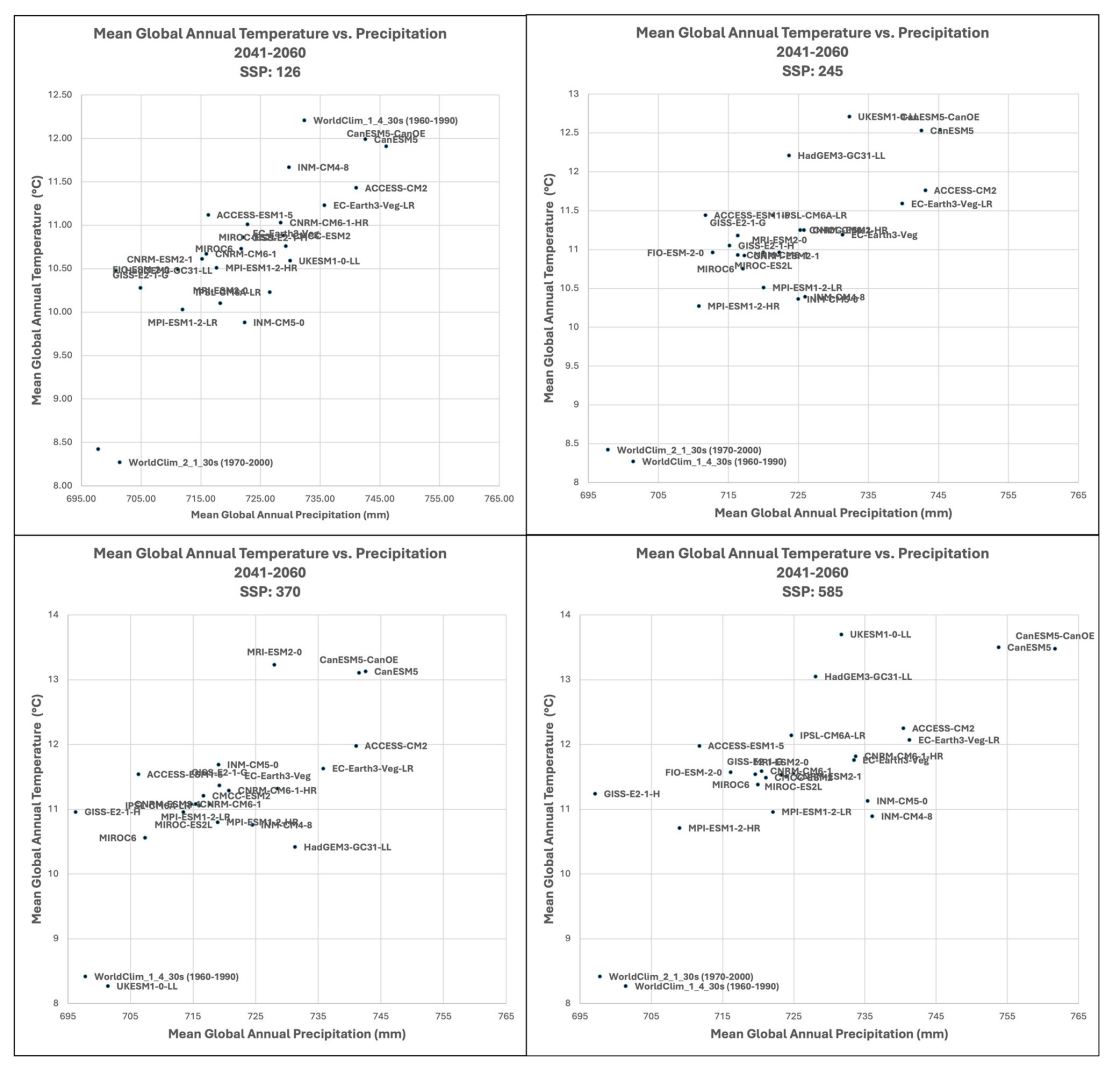
Distribution of 22 CMIP6 Earth System Models along temperature and precipitation gradients (2041–2060). Distribution of average global mean annual temperature and mean annual precipitation of 22 CMIP6 ESM for four SSP for the time period 2041–2060.

Based upon the distribution of the various scenarios along their projected temperature and precipitation estimates for the 2021–2040 future time period ( Figure 3 & Figure 4), three multi-model ensembles, each articulated by their four respective SSPs, were identified ( Table 1).

- **All Models:** includes all of the 22 ESM, as available within a particular SSP.
- **High Risk:** includes 5 ESM identified as projecting the highest increases in temperature and precipitation and lying outside and significantly higher than the majority of estimates.
- Majority **Consensus**: includes 15 ESM, that is, all available ESM excluding the ESM in the “High Risk” category, and those missing data across all of the 4 SSP. Further herein referred to as the “Consensus” category.

The three parameters of monthly minimum temperature, monthly maximum temperature and monthly precipitation for ESM’s included within each of these ensembles were averaged for each of their respective SSPs. These averaged parameters were then used to calculate average PET and AI for each ensemble category as per the above methodology.

**Figure 5.**
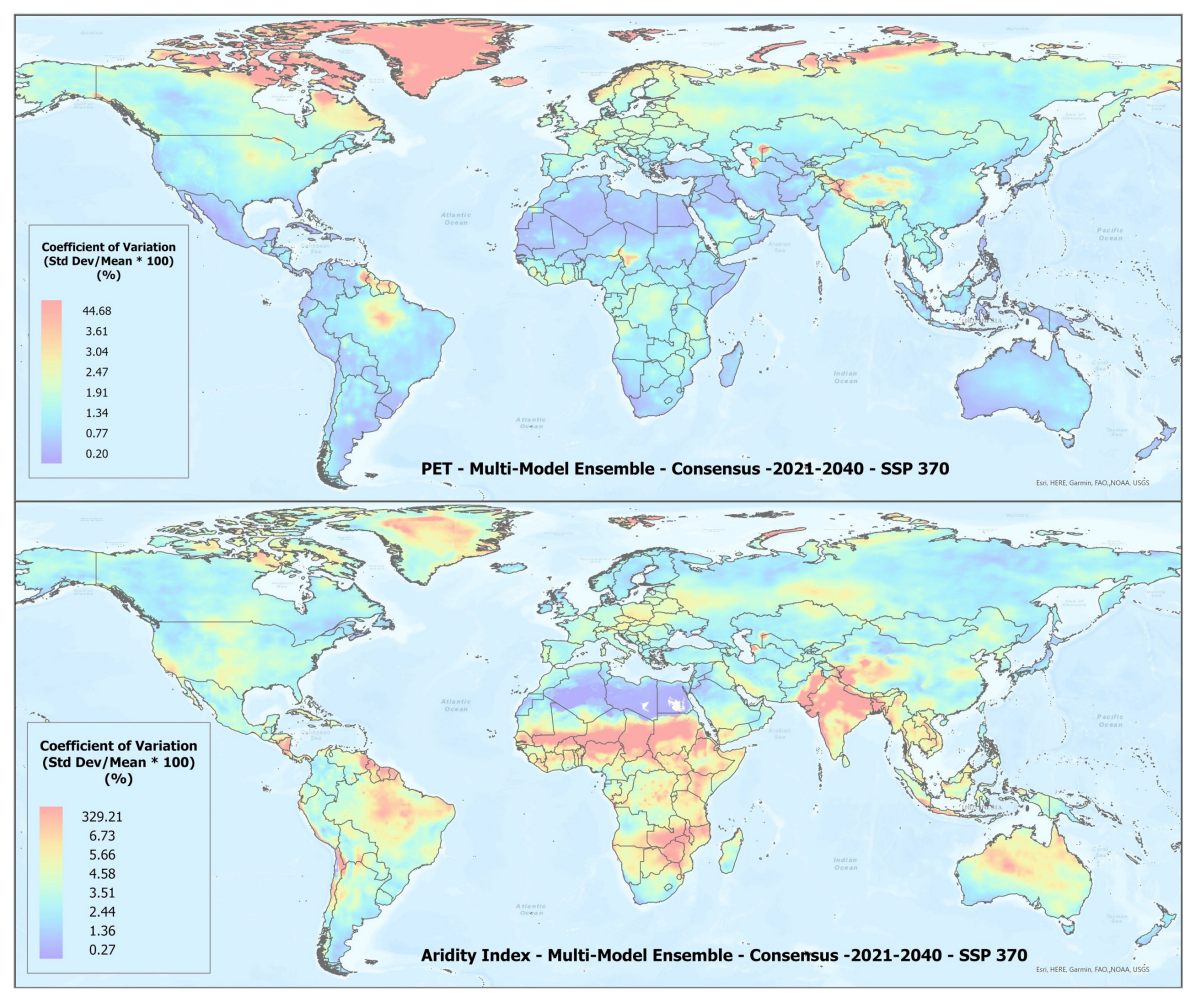
Example of spatial variation of Coefficient of Variation (CoV) of PET and Aridity Index. Coefficient of variation of PET and Aridity Index as shown for the “Consensus” multimodel ensemble average, SPP 370 scenario, shown here to illustrate variation in agreement across the various global regions.

In general, there was found to be a high level of agreement amongst the ESM included within each of the respective averaged multimodel ensembles ( Table 4), however, as illustrated in Figure 5, with significant variability across regions (See Extended Materials for regional results). Agreement was fairly high for the basic parameters of annual average temperature and annual average precipitation with all values below 5%, indicating very low variability relative to the mean, suggesting strong consistency or agreement. Somewhat less for annual averaged PET and AI, which is slightly increased in the 2041–2060 time period. To compensate for negative values, the coefficient of variation (CoV) for mean annual temperature was calculated by adding 33 degrees to the annual temperature value.

**Table 4.** Coefficient of variation of ensemble averages. Coefficient of variation showing agreement amongst CMIP_6 Earth System Models (ESM) included with each of three averaged multimodel ensembles, as articulated for each of four shared socio-economic pathways (SSP: Scenario).

MultiModel Ensemble		Coefficient of Variation (%)			
		2021–2040			
	Scenario	tmean_yr	prec_yr	pet_yr	aridity_index
**All**	126	2.08	4.24	2.95	5.30
	245	2.21	4.42	3.19	5.68
	370	2.49	4.96	3.48	6.36
	585	2.56	5.32	3.85	7.07
	**Ensemble average:**	**2.34**	**4.74**	**3.37**	**6.10**
**Consensus**	126	1.44	3.64	2.37	4.75
	245	1.44	3.65	2.37	4.83
	370	1.36	3.85	2.33	5.12
	585	1.48	4.31	2.61	5.73
	**Ensemble average:**	**1.43**	**3.86**	**2.42**	**5.11**
**High Risk**	126	1.09	2.63	1.71	3.72
	245	1.17	2.68	1.75	3.80
	370	1.30	2.61	1.69	3.58
	585	1.22	2.88	1.71	3.91
	**Ensemble average:**	**1.20**	**2.70**	**1.72**	**3.75**
MultiModel		2041–2060			
Ensemble	Scenario	tmean_yr	prec_yr	pet_yr	aridity_index
**All**	126	2.08	4.19	2.94	5.24
	245	2.21	4.42	3.19	5.67
	370	2.49	4.00	2.42	5.18
	585	2.56	4.45	2.69	5.78
	**Ensemble average:**	**2.34**	**4.27**	**2.81**	**5.47**
**Consensus**	126	1.44	3.70	2.41	4.70
	245	1.44	3.82	2.46	4.90
	370	1.36	4.03	2.44	5.19
	585	1.48	4.49	2.71	5.79
	**Ensemble average:**	**1.43**	**4.01**	**2.51**	**5.15**
**High_Risk**	126	1.25	3.18	2.17	4.67
	245	1.34	3.47	2.40	5.14
	370	1.72	3.78	2.80	5.66
	585	1.76	4.32	3.04	6.42
	**Ensemble average:**	**1.52**	**3.69**	**2.60**	**5.47**

**Figure 6.**
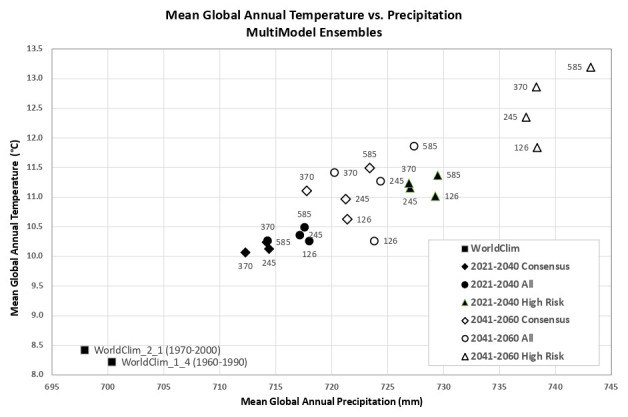
Distribution of historical and projected global averaged annual temperature and annual precipitation. Distribution of historical and projected global averaged annual temperature (°C) and annual precipitation (mm) of three averaged multimodel ensembles, across four shared socio-economic pathways (SSP: 126; 245; 370; 585).

**Figure 7.**
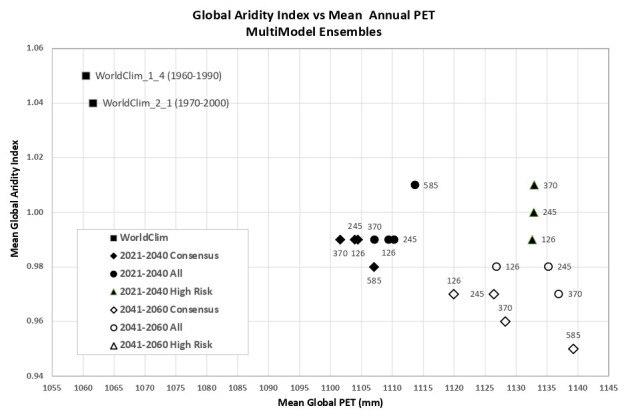
Distribution of historical and projected global averaged PET and Aridity Index. Distribution of historical and projected global averaged PET (mm) and the aridity index (AI) of three averaged multimodel ensembles, across four shared socio-economic pathways (SSP: 126; 245; 370; 585). Higher AI values indicate wetter conditions, lower values indicate drier conditions.

An overview of the distribution of the global average annual temperature and average annual precipitation projected by the various ensembles, as articulated to each SSP ( Figure 6) illustrates that all models indicate global trends of increasing precipitation and increasing temperature in the near-term, more so by the 2041–2060 time period. However, when considering the projected global increase in PET ( Figure 7), there emerges a distinct and significant drying trend indicated by decreasing AI values, both in the near-term, and again, more so by 2041–2060. There is, however, significant variability shown among regions (See Extended Data for tabular and regional descriptive statistics).

## Technical validation

**Figure 8.**
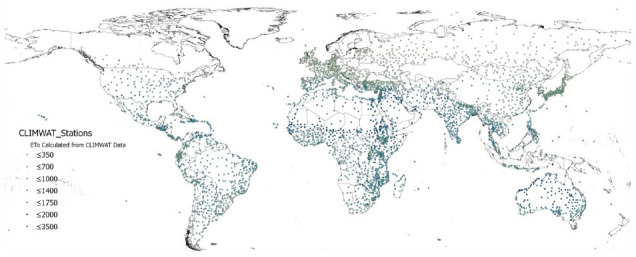
Location of weather stations included in the FAO CLIMWAT dataset. Location of weather stations included in the FAO CLIMWAT dataset, showing ET0_CLIMWAT values for Penman-Monteith Reference Evapotranspiration (ET0) ( Muñoz & Greiser, 2006).

The global estimations of PET (Hargreaves method) and ET _0_ (Penmen-Monteith), and their respective AI derived from these, were evaluated against the FAO “CLIMWAT 2.0 for CROPWAT” ( Muñoz & Greiser, 2006) global climate database using long-term monthly mean values of climatic parameters derived from weather station data, roughly covering the period of 1971–2000, concurrent with the temporal coverage of the WorldClim version 2.1 database ( Fick & Hijmans, 2017; Zomer *et al.*, 2022). CLIMWAT 2.0 provides observed agroclimatic data of over 5000 stations distributed worldwide ( Figure 8), including monthly averages for seven climatic parameters, namely maximum temperature, minimum temperature, relative humidity, wind speed, sunshine hours, radiation balance and ET _0_ calculated according to the Penman-Monteith method, as well as the coordinates and altitude of the station. After evaluation, 4242 stations were used for the spreadsheet analysis, and a subset of data points common among all geospatial datasets created, which came out to 3842 stations.

Input parameters for validation were extracted from each of the two historical geospatial datasets by sampling of the gridded data at the weather station coordinates and were compared with the values from the weather station data to evaluate the accuracy and overlap of the CLIMWAT and WorldClim datasets, and the suitability of using the CLIMWAT to evaluate the performance of the PET spatial estimation. An assessment of the input parameters from the WorldClim datasets showed high levels of correspondence when compared to the CLIMWAT data ( Zomer *et al.*, 2022) that the CLIMWAT was considered an appropriate dataset available for evaluating the accuracy of the PET and AI estimation algorithms.

**Figure 9.**
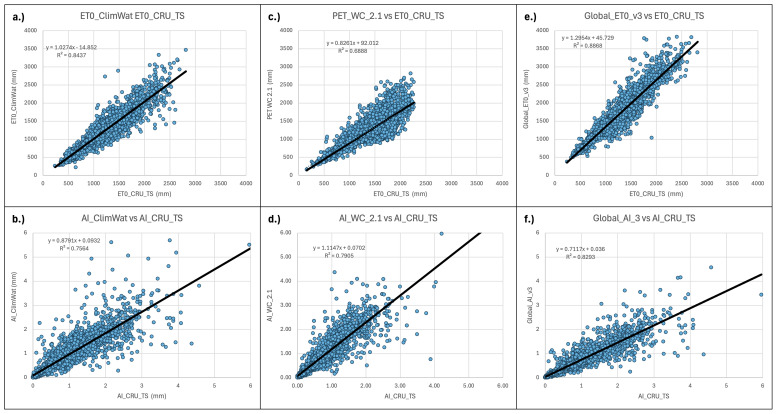
Validation and comparison of input data used in the analysis and results. Validation and comparison of: **a**.) PET (Hargreaves) calculated from the CLIMWAT ( Muñoz & Greiser, 2006) weather station data versus the CLIMWAT reported ET _0_ (Penman-Monteith); **b**.) ET _0_ (Penman-Monteith) calculated from the CLIMWAT weather station data versus the CLIMWAT reported ET _0_ (Penman-Monteith); **c**.) PET Hargreaves (WorldClim 2.1) versus CLIMWAT reported ET _0_ (Penman-Monteith); **d**.) Aridity Index ((WorldClim 2.1) versus Aridity Index calculated from CLIMWAT weather station data and reported ET _0 _(Penman-Monteith); **e**.) PET (Hargreaves) versus Global ET _0_ – Version 3 ET _0_ (Penman-Monteith).

The previously developed dataset, Global-AI_PET_v3 ( Zomer *et al.*, 2022), based upon WorldClim v. 2.1 and calculated using the Penman-Monteith equation was used as a comparative standard, with the assumption that the full implementation of the Penman-Monteith, it being the standard, would provide the more robust results. PET estimates were tested and compared both using the Hargreaves and the Penman-Monteith equations with CLIMWAT provided parameters from 4242 weather stations to parameterize the estimation algorithm ( Table 5; Figure 9). The calculated values for the Hargreaves equation were shown to have a moderate level of correspondence (r ^2^ = 0.68) with a fairly high standard error (346 mm). As expected, the Penman-Monteith results were highly accurate (r ^2^ = 0.99; standard error = 36mm), as they replicated the CLIMWAT weather station reported results, i.e., using the same equation, confirming the relative accuracy of the dataset and its fitness for use as a comparative standard. The algorithm was then implemented geospatially on a per grid cell basis to produce global geospatial datasets calculated based upon the Hargreaves equation for the two historical time periods (WorldClim 1.4 – 1960–1990; WorldClim 2.1 – 1970–2000) and tested against the CLIMWAT ET _0_ estimates from 3842 weather stations (i.e. all weather stations with useable data across all of the compared datasets). Although the results showed a higher level of accuracy (r ^2^ = 0.85) for the Penman-Monteith calculated dataset (Global_ET _0__v3), the relatively (or moderately) high accuracy (r ^2^ = 0.72) attained by the Hargreaves implementation of the algorithm maybe considered sufficient for use within many modeling and other efforts, however, with caveats in light of the variability associated with both the input data and accuracy of the estimation algorithm when applied globally. In particular, local estimates may have high variability associated with steep elevation gradients and heterogenous terrain, and/or low levels of accuracy at the grid cell level in certain regions due to interpolation of scattered or less dense weather station data. There is significant potential for error associated with both the interpolated and downscaled global input data and the application of the Hargreaves methodology (or Penman-Monteith for that matter) when implemented globally. It has been noted that the Hargreaves equation tends to under-estimate ET _o_ under high wind conditions (> 3m/s) and to over-estimate under conditions of high relative humidity ( Richards, 2015).

**Table 5.** Summary Table of Technical Validation Results.

Regression	R Square	Standard Error	Bias
**Potential Evapotranspiration_ETo** PET_CLIMWAT_XLS vs ET _o__CLIMWAT ET _0__CLIMWAT_XLS vs ET _0__CLIMWAT PET_WC_1.4 vs ETo_CLIMWAT PET_WC_2.1 vs ET _0__CLIMWAT Global_ET _0__v3 vs ET _0__CLIMWAT PET_WC_2.1 vs Global_ET _0__v3 Global_ET _0__v3 vs ET _0__CRU_TS	0.680 0.99 0.72 0.76 0.85 0.67 0.89	345 36 221 212 219 238 136	586.30 -53.71 -132.65 -128.90 -389.38 -262.12 -408.12
**Aridity Index** AI_WC_1.4 vs AI_CLIMWAT AI_WC_2.1 vs AI_CLIMWAT Global_AI_v3 vs AI_CLIMWAT AI_WC_2.1 vs Global_AI_v3 AI_CLIMWAT vs AI_CRU_TS AI_WC_2.1 vs AI_CRU_TS Global_AI_v3 vs AI_CRU_TS	0.91 0.88 0.90 0.89 0.77 0.79 0.83	0.16 0.18 0.17 0.18 0.33 0.26 0.22	0.14 0.14 0.21 0.07 -0.02 -0.16 -0.23
** Evaluated datasets	Description		
**PET (ET _0_)** ET _0__ClimWat ET _0__ClimWat_XLS ET _0__CRU_TS PET_WC_1.4 PET_WC_2.1 Global_ET _0__v3	ET _0_ as reported by CLIMWAT station data ET _0_ calculated using estimation algorithm parameterized with CLIMWAT station data ET _0_ extracted from CRU_TS PET grid PET calculated using WC_1.4 1960 - 1990 (Modified Hargreaves-Thornton) PET calculated using WC_2.1 - 1970 - 2000 (Modified Hargreaves-Thornton) ET _o_ calculated using WC_2.1 - 1970 - 2000 (Penman-Monteith)		
**Aridity Index (AI)** AI_ClimWat AI_CRU_TS AI_WC_1.4 AI_WC_2.1 Global_AI_v3	AI calculated using parameters from CLIMWAT station data - 1960-1990 (Penman-Monteith) AI calculated using ET _0__CRU_TS - 1970-2000 (Penman-Monteith) AI calculated using WC_1.4 - 1990 - 2000 (Modified Hargreaves-Thornton) AI calculated using WC_2.1 - 1970 - 2000 (Modified Hargreaves-Thornton) AI calculated using WC_2.1 - 1970 - 2000 (Penman-Monteith)		

There were significant differences between the Hargreaves (PET_WC_2.1) and Penman-Monteith (Global_ET0_v3) versions of the global implementation (r ^2^ = 0.67). PET_WC_2.1 using the Hargreaves methodology appeared to systematically underestimate higher PET values. Most of these higher values were generally outside of the prescribed realm of validity for the PET estimates related to plant growth or agricultural activities, and mostly found in the higher latitudes and arctic regions. However, the AI estimates based on the AI_WC_2.1 analysis, when compared to AI estimates based on parameters provided by the CLIMWAT weather station data ( Table 5; Figure 9e), showed a high level of correspondence (r ^2^ = 0.91), statistically the same as that from the Global-AI_PET_v3 estimates (r ^2^ = 0.90).

**Figure 10.**
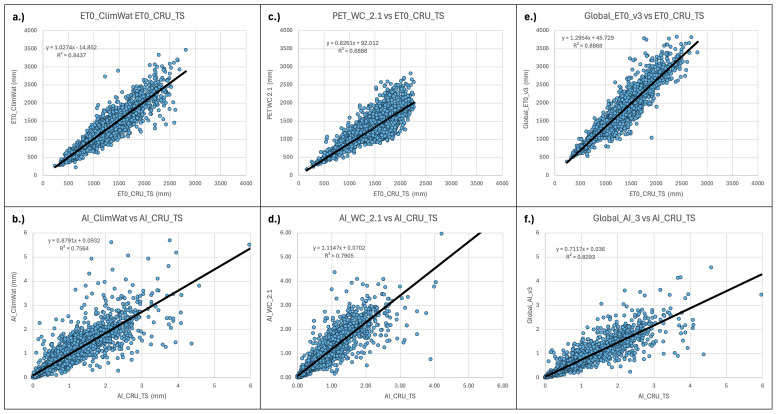
Validation and comparison of Hargreaves PET and Aridity Index vs. CRU_TS dataset. Validation and comparison of results for the PET (Hargreaves) and the Aridity Index datasets with the CRU_TS ( Harris *et al.*, 2020) dataset.

Similarly, the estimations of the Hargreaves PET (PET_WC_2.1) and ET _0_ (Global_ET _0__v3) were evaluated against the ET _0_ dataset provided by the CRU_TS (Climatic Research Unit gridded Time Series version 4.05) ( Harris *et al.*, 2020). The CRU_TS is a widely used climate dataset on a 0.5° latitude by 0.5° longitude grid over all land domains of the world except Antarctica. It is derived by the interpolation of monthly climate anomalies from extensive networks of weather station observations. PET values are provided in the CRU_TS dataset, calculated based upon the Penman-Monteith formula ( Droogers & Allen, 2002; Zomer *et al.*, 2022; Zotarelli *et al.*, 2018), using the CRU_TS gridded values of mean temperature, vapour pressure, cloud cover and wind field. For our comparison, we averaged the CRU_TS monthly values for PET from 1971–2000 to obtain a global coverage of average annual PET for that time period. The same CLIMWAT meteorological stations used in previous comparisons were used as sample points for the comparison with both the PET and ET _0_ datasets, and the CLIMWAT ET _0_ was also compared with the CRU_TS PET (ET _0__CRU_TS) dataset (r ^2^ = 0.84) to assess general congruence among the datasets ( Table 5; Figure 10). Results showed a moderate level of agreement (r ^2^ = 0.68) between the ET _0__CRU_TS and Hargreaves (PET_WC_2.1) data, with a significantly higher level of agreement, as expected, for the Penman-Monteith ((Global_ET _0__v3: r ^2^ = 0.89)) results. However, both the Hargreaves and the Penman-Monteith calculated aridity indices showed high correspondence to each other and to the CLIMWAT and CRU_TS results. The CRU_TS precipitation data for the 1971–2000 time period was similarly averaged and used to calculate an AI based upon the CRU_TS dataset and compared to both the PET_WC_2.1 and the Global_AI_v3. The Hargreaves calculated geospatial aridity index dataset (AI_WC_2.1) showed high correspondence (r ^2^= 0.88) with the aridity index results calculated from the CLIMWAT station data (AI_CLIMWAT), only slightly less than the Global_AI_v3 (r ^2^ = 0.90). Both the AI_WC_2.1 and Global_AI_3 performed well (respectively r ^2^ = 0.79 and r ^2^ = 0.83) against the CRU_TS calculated AI (AI_CRU_TS).

Among other factors, the significant and specific influence of relative humidity and wind speed lead to particular deviations of results across broad climate regions between Hargreaves and Penman-Monteith model results ( Allen *et al.*, 1998). This uncertainty has led to several successful attempts ( Aschonitis *et al.*, 2017; Tegos *et al.*, 2017) to provide calibration and correction factors map for Hargreaves estimates, based on parametric regression between Hargreaves PET and PM ET0 monthly values for historical condition, that can close the gap between results from Hargreaves and PM methods. While recognizing the value of such efforts, expansion on the use of these parametric corrections have still been quite limited on future projections, This would require at any site not basic changes in correlation between temperature (used in Hargreaves equations) with the others variables (RH, wind speed, solar radiation) additionally used in the Penman-Monteith equation, to maintain relative divergence between Hargreaves and Penman-Monteith values steady (and in turn correction coefficients) from historical to future conditions.

It is the authors conclusion given the high levels of uncertainty ( Wang *et al.*, 2015) and the variability associated with future projections and interpolated climate datasets in general, that these results can provide meaningful inputs and useful insights regarding both trends and magnitude of change, but of course, should be used keeping in mind this uncertainty.

## Usage notes

The geospatial datasets are provided online in GeoTIFF (.tif) format, in geographic coordinates; datum and spheroid are WGS84; spatial units are decimal degrees. The spatial resolution is 30 arc-seconds or 0.008333 degrees (approximately 1 km ^2^ at the equator). The PET values are defined as total mm of PET per month or per year. The Aridity Index values are unitless.

Although considered sufficient for use within many modeling and other scientific efforts, we highlight the earlier caveats showing the variability associated with both the input data and accuracy of the estimation algorithm when applied globally. As stated, local or even regional estimates may have high variability associated with steep elevation gradients and heterogenous terrain, and/or low levels of accuracy at the grid cell level due to interpolation of scattered or less dense weather station data, with significant potential for error associated with both the global input data ( Navarro *et al.*, 2024) and the application of the Hargreaves methodology ( Wang *et al.*, 2015) when implemented globally across a wide spectrum of environmental, geophysical, and bioclimatic conditions. As such, while useful for identifying and determining change, we urge caution in use of these datasets. Likewise, the aridity index, and the Hargreaves equation, are geared toward ascertaining moisture available for crop or other plant growth, and as such may not be designed for biomes and bioclimates beyond the reasonable temperature limits of seasonal agriculture. Aridity Index provides estimate on annual temporal scale, while other methods (e.g. SPEI, PSDI) can be further implemented using these PET and WorldClim datasets to derive other specific agrometeorological drought indicators at monthly and seasonal scale ( Tegos *et al.*, 2023; Vicente-Serrano *et al.*, 2010). Some very high values of Aridity Index, primarily found in the highest latitudes and arctic regions, although remaining in the dataset, are considered uninterpretable. 

## Ethics and consent

Ethical approval and consent were not required.

## Data Availability

The Future_Global_AI_PET data layers have been processed and finalized for distribution online as GEO-TIFFs. These datasets have been zipped (.zip) into monthly series or their respective annual layers, by each combination of climate model and SSP scenario. The Future_Global_AI_PET Database is archived and available online from the Science Data Bank (ScienceDB, at https://doi.org/10.57760/sciencedb.nbsdc.00086 ( Zomer & Trabucco, 2024). The “Future_Global_AI_PET Database” provides a set of high-resolution (30 arc-seconds) global raster datasets of average monthly and annual potential evapotransipation (PET) and annual aridity index (AI) for two historical (1960–1990; 1970–2000) and two future (2021–2040; 2041–2060) time periods for each of 25 CMIP6 Earth System Models across four emission scenarios (SSP: 126, 245, 370, 585). The database also includes three averaged multi-model ensembles produced for each of these four emission scenarios. There are a total of 389 files online, including a detailed Readme file, comprising 389 GB of data. The Future_Global_AI_PET data layers have been processed and finalized for distribution online as GEO-TIFFs. These datasets have been zipped (.zip) into monthly series or their respective annual layers, by each combination of climate model and SSP scenario. The Future_Global_AI_PET Database is archived and available online from the Science Data Bank (ScienceDB), at https://doi.org/10.57760/sciencedb.nbsdc.00086 ( Zomer & Trabucco, 2024). Supplementary tables and figures are available online on the Figshare Data Repository as “Extended Data: CMIP6-Based Global Estimates of Future Aridity Index and Potential Evapotranspiration for 2021–2060” at https://doi.org/10.6084/m9.figshare.26095363 ( Zomer *et al.*, 2024). These are available under a CC-BY 4.0 license in one file (Global Future AI_PET - SM_Tables v1) containing supplementary tables and a figure including: Global Mean Annual Climatic Parameters for All Included CMIP_6 ESM Mean Annual Climatic Parameters for all Multi-model Ensembles by Region Coefficient of Variation for all Multi-model Ensembles by Region Map of Global Regions used in the Regional Analysis

## Code availability

The algorithm used to calculate PET and AI was coded in Python 3.2 and uses the ArcPy (ArcGIS) package. It is available under a Apache 2.0 license on Figshare at this link below:

https://figshare.com/articles/software/Global_Future_PET_AI_Algorithm_Code_Python_-_Calculate_PET_AI/24978666
