# Supplementary material for: CMIP6-based global estimates of future aridity index and potential evapotranspiration for 2021-2060
Source: Open Res Eur. 2025 May 12;4:157. Originally published 2024 Jul 29. [Version 4] doi: 10.12688/openreseurope.18110.4 (PMC11806256; doi:10.12688/openreseurope.18110.4)
Supplement: Peer review history

# Reviewer report: Reviewer response for version 4

**DOI:** 10.21956/openreseurope.22030.r54138

**Published:** 2025-05-23

**Authors:** Vassilis Aschonitis

**Affiliations:** 1 Hellenic Agricultural Organization, Soil and Water Resources Institute, Thessaloniki, Greece

no new comments

Are sufficient details of methods and materials provided to allow replication by others?

Yes

Is the rationale for creating the dataset(s) clearly described?

Yes

Are the datasets clearly presented in a useable and accessible format?

Yes

Are the protocols appropriate and is the work technically sound?

Yes

Reviewer Expertise:

agrometeorology

I confirm that I have read this submission and believe that I have an appropriate level of expertise to confirm that it is of an acceptable scientific standard.

# Reviewer report: Reviewer response for version 3

**DOI:** 10.21956/openreseurope.21447.r51854

**Published:** 2025-03-27

**Authors:** Louise Crochemore

**Affiliations:** 1 nstitute of Environmental Geosciences - CNRS, INRAE, IRD, Grenoble-INP, Université Grenoble Alpes, Grenoble, France

We thank the authors for their thorough answers, and do acknowledge that this is a data note and not a research article. As such, our points were well addressed. Here are a few remaining recommendations:

  - Please check again the spelling of Penman-Monteith throughout the paper.

  - The reference to Wang et al. (2018) for extraterrestrial radiation still seems erroneous. Please check. Neither the words "radiation" nor "extraterrestrial" appear in this article. The article seems to rather focus on transfers in porous media. Wang X, et al., (Ref 1)

  - The information mentioned by the authors on the choice of the High risk scenarios would be useful to add to the note.

  - Similarly, the information on why the number of CLIMWAT stations varies throughout the note is useful. Consider adding it at the beginning of the Technical validation.

Are sufficient details of methods and materials provided to allow replication by others?

Partly

Is the rationale for creating the dataset(s) clearly described?

Yes

Are the datasets clearly presented in a useable and accessible format?

Yes

Are the protocols appropriate and is the work technically sound?

Partly

Reviewer Expertise:

NA

I confirm that I have read this submission and believe that I have an appropriate level of expertise to confirm that it is of an acceptable scientific standard.

## References

[1] : Performance of HYDRUS-1D for simulating water movement in water-repellent soils. *Canadian Journal of Soil Science* .2018;98(3) : 10.1139/cjss-2017-0116 407-420 10.1139/cjss-2017-0116

# Response

**Published:** 2025-05-01

**Authors:** Robert Zomer

**Affiliations:** IAFES, Foundation Euro-Mediterranean Center for Climate Change, Sassari, Sardinia, Italy

Thanks again to the Reviewers for greatly improving the manuscript...   we have gone back and now addressed final points: 1.) Corrected Spellin:   Penman-Monteith (2 instances found)

 2.) Corrected Reference:   Yes, .. the Wang et al., 2018, was incorrect,.. mislabelled in our bibliographic software.  Correction citation is: 

 Šimůnek, J., M. Th. van Genuchten and M. Šejna, The HYDRUS-1D Software Package for Simulating the Movement of Water, Heat, and Multiple Solutes in Variably Saturated Media, Version 3.0, HYDRUS Software Series 1, Department of Environmental Sciences, University of California Riverside, Riverside, California, USA, 2005.

 3.) Added this statement to Methods:  "After evaluation, 4242 stations were used for the spreadsheet analysis, and a subset of data points common among all geospatial datasets created, which came out to 3842 stations."

 4.) Corrected this statement in Methods: " Based upon the distribution of the various scenarios along their projected temperature and precipitation estimates *for the 2021-2040 future time period* ( Figure 3 & Figure 4), three multi-model ensembles, each articulated by their four..."

# Reviewer report: Reviewer response for version 2

**DOI:** 10.21956/openreseurope.21154.r50881

**Published:** 2025-02-08

**Authors:** Aristoteles Tegos

**Affiliations:** 1 National Technical University Athens, Athens, Greece

The authors have taken on board my comments and the manuscript has been substantially improved.

Are sufficient details of methods and materials provided to allow replication by others?

Partly

Is the rationale for creating the dataset(s) clearly described?

Partly

Are the datasets clearly presented in a useable and accessible format?

Partly

Are the protocols appropriate and is the work technically sound?

Partly

Reviewer Expertise:

Potential Evapotranspiration expert. Water Resources Management and hydrology epxert.

I confirm that I have read this submission and believe that I have an appropriate level of expertise to confirm that it is of an acceptable scientific standard.

# Reviewer report: Reviewer response for version 2

**DOI:** 10.21956/openreseurope.21154.r50879

**Published:** 2025-02-07

**Authors:** Vassilis Aschonitis

**Affiliations:** 1 Hellenic Agricultural Organization, Soil and Water Resources Institute, Thessaloniki, Greece

I think the authors improved the document. Just some minor corrections:

 1. Check the numbering of equations

 2. The coefficient in Hargreaves equation is 0.0023 and not 0.023.

Are sufficient details of methods and materials provided to allow replication by others?

Yes

Is the rationale for creating the dataset(s) clearly described?

Yes

Are the datasets clearly presented in a useable and accessible format?

Yes

Are the protocols appropriate and is the work technically sound?

Yes

Reviewer Expertise:

Hydrology, Hydroclimatology, Soil Science, Environmental Modelling

I confirm that I have read this submission and believe that I have an appropriate level of expertise to confirm that it is of an acceptable scientific standard.

# Reviewer report: Reviewer response for version 1

**DOI:** 10.21956/openreseurope.19576.r43858

**Published:** 2024-09-27

**Authors:** Aristoteles Tegos

**Affiliations:** 1 National Technical University Athens, Athens, Greece

The manuscript presents a high-resolution average annual and monthly global estimates of potential evapotranspiration (PET) and aridity index (AI) for 22 CMIP6 Earth System Models for two future (2021-2041; 2041-2060) and two historical (1960-1990; 1970–2000) time periods. The paper is well written but needs several modifications to include recent scientific findings.

 My main concern is the lack of the discussion for the required local calibration of Hargreaves model which has been already  proved to be necessary given the physical complexity of PET phenomenon (Tabari and Talaee 2011( Ref 1), Haslinger and Bartsc 2016  (Ref 2)). The same, using the same global database, has been highlighted by Tegos et al. 2017  (Ref 3)  where the local calibration of temperature-base or simplified extraterrestrial are  irreplaceable for improving the performance of simplified PET approaches (also Tegos et al 2015 (Ref 4)). I would recommend further discussion on that in order to provide limitations of the current dataset and also future improvements especially in geographic where temperature-radiation approaches are failed to provide reliable PET estimates such as Amazonios etc (Tegos et al .2017 (Ref 5))

*Among the various equations employed for estimating PET.* Suggest to add as citation the paper which presents a detailed review of PET model (McMahon et al 2016).

*The Hargreaves ( Hargreaves, 1994b) equation demonstrated the nearest results to the classic PM ( Droogers & Allen, 2002; Itenfisu et al., 2003). The Hargreaves method with substantially less data requirements, i.e., requiring only measured values of maximum and minimum temperatures and precipitation, was recommended for more general use and therefore is considered more appropriately applicable to data scarce regions and situations ( Droogers & Allen, 2002). For the purposes of this paper, to maintain clarity, we refer to reference evapotranspiration calculated using the Penman-Monteith as “ET _0_” and calculations using the Hargreaves equation as “PET”. *Please refer to my above comment on the historical criticism of the uncalibrated Hargreaves model. The txt must provide more details in the point. 

 There is a need for additional discussion of Aridity index (Vicente-Serrano et al. 2010 (Ref 6)). The original index was based on the use of the PET Thornthwaite model however recent a research has been criticized the use Thornthwaite model for assessing aridity severity (Tegos et al. 2023 (Ref 7)) and other PET models are recommended.

Are sufficient details of methods and materials provided to allow replication by others?

Partly

Is the rationale for creating the dataset(s) clearly described?

Partly

Are the datasets clearly presented in a useable and accessible format?

Partly

Are the protocols appropriate and is the work technically sound?

Partly

Reviewer Expertise:

Potential Evapotranspiration expert. Water Resources Management and hydrology epxert.

I confirm that I have read this submission and believe that I have an appropriate level of expertise to confirm that it is of an acceptable scientific standard, however I have significant reservations, as outlined above.

## References

[1] : Local Calibration of the Hargreaves and Priestley-Taylor Equations for Estimating Reference Evapotranspiration in Arid and Cold Climates of Iran Based on the Penman-Monteith Model. *Journal of Hydrologic Engineering* .2011;16(10) : 10.1061/(ASCE)HE.1943-5584.0000366 837-845 10.1061/(ASCE)HE.1943-5584.0000366

[2] : Creating long term gridded fields of reference evapotranspiration in Alpine terrain based on a re-calibrated Hargreaves method.2015; 10.5194/hessd-12-5055-2015 10.5194/hessd-12-5055-2015

[3] : Parametric Modelling of Potential Evapotranspiration: A Global Survey. *Water* .2017;9(10) : 10.3390/w9100795 10.3390/w9100795

[4] : A parsimonious regional parametric evapotranspiration model based on a simplification of the Penman–Monteith formula. *Journal of Hydrology* .2015;524: 10.1016/j.jhydrol.2015.03.024 708-717 10.1016/j.jhydrol.2015.03.024

[5] : Historical developments of models for estimating evaporation using standard meteorological data. *WIREs Water* .2016;3(6) : 10.1002/wat2.1172 788-818 10.1002/wat2.1172

[6] : A Multiscalar Drought Index Sensitive to Global Warming: The Standardized Precipitation Evapotranspiration Index. *Journal of Climate* .2010;23(7) : 10.1175/2009JCLI2909.1 1696-1718 10.1175/2009JCLI2909.1

[7] : On the Sensitivity of Standardized-Precipitation-Evapotranspiration and Aridity Indexes Using Alternative Potential Evapotranspiration Models. *Hydrology* .2023;10(3) : 10.3390/hydrology10030064 10.3390/hydrology10030064

# Response

**Published:** 2025-01-29

**Authors:** Robert Zomer

**Affiliations:** IAFES, Foundation Euro-Mediterranean Center for Climate Change, Sassari, Sardinia, Italy

**Reviewer Report - 3** 2 Views

**Approved with reservations**

**27 Sep 2024** VERSION 1

**Aristoteles Tegos**, National Technical University Athens, Athens, Greece  

**Reviewer Comments:**

 The manuscript presents a high-resolution average annual and monthly global estimates of potential evapotranspiration (PET) and aridity index (AI) for 22 CMIP6 Earth System Models for two future (2021-2041; 2041-2060) and two historical (1960-1990; 1970–2000) time periods. The paper is well written but needs several modifications to include recent scientific findings.

 My main concern is the lack of the discussion for the required local calibration of Hargreaves model which has been already  proved to be necessary given the physical complexity of PET phenomenon (Tabari and Talaee 2011( Ref 1), Haslinger and Bartsc 2016  (Ref 2)). The same, using the same global database, has been highlighted by Tegos et al. 2017  (Ref 3)  where the local calibration of temperature-base or simplified extraterrestrial are  irreplaceable for improving the performance of simplified PET approaches (also Tegos et al 2015 (Ref 4)). I would recommend further discussion on that in order to provide limitations of the current dataset and also future improvements especially in geographic where temperature-radiation approaches are failed to provide reliable PET estimates such as Amazonios etc (Tegos et al .2017 (Ref 5)).

**Author Response:**

 We do reckon the added value of parametric corrections for the Hargreaves equations, in order to reduce gap with PM Monteith and especially some specific climate broader areas. The global database of corrected parametric equations for the Hargreaves eq (Tegos et al. 2017) can provide valuable information to improve accuracy of the Hargreaves method in such regions. Tegos et al. (2017) provide a spatial distribution of corrected parametric coefficients, based on regression between Hargreaves PET and PM ET0 monthly values for historical periods (based on historical observation available from CLIMWAT 2.0 database). As mentioned also by the first reviewer, several studies have tested calibration of Hargraves for current climate conditions. While recognizing the value of such efforts, we can not yet assume that such parametric regression (and thereafter the parameter correction) hold steady at same location in the far future (up to 2060). This would imply at any site not basic changes in the correlation between temperature (used in Hargreaves equations) with the others variables (RH, Wind Speed, solar radiation) additionally used in the PM equation, to maintain relative divergence between Hargreaves and Penman-Monteith values steady (and in turn correction coefficients) from historical to future conditions. In fact, none of the mentioned citations (Tabari and Talaee 2011, Haslinger and Bartsc 2016, Tegos et al. 2017, Tegos et al 2015) uses corrected parameters factors to climate projections. Valuable effort to improve use of correction coefficient for future climate conditions, could possibly define correction factors that would be climate explicit (possibly in relation to Temperature regimes), rather than just spatially explicit.  Any shift in the climate regime, due to climate change, would be associated with the shift of corrected parameters. To Acknowledge the value of corrected parameters to improve reliability of Hargreaves equation, we have added the following in technical validation section *Among other factors, the significant and specific influence of relative humidity and wind speed lead to particular deviations of results across broad climate regions between Hargreaves and Penman-Monteith model results (Allen et al., 1998). This uncertainty has led to several successful attempts (Tegos et al. 2017,* *Aschonitis et al. 2017) to provide calibration and correction factors map for Hargreaves estimates, based on parametric regression between Hargreaves PET and PM ET0 monthly values for historical condition, that can close the gap between results from Hargreaves and PM methods.  While recognizing the value of such efforts, expansion on the use of these parametric corrections have still been quite limited on future projections, This would require at any site not basic changes in correlation between temperature (used in Hargreaves equations) with the others variables (RH, Wind Speed, solar radiation) additionally used in the PM equation, to maintain relative divergence between Hargreaves and Penman-Monteith values steady (and in turn correction coefficients) from historical to future conditions.*

**Reviewer Comments:**

*Among the various equations employed for estimating PET.* Suggest to add as citation the paper which presents a detailed review of PET model (McMahon et al 2016).

**Author Response:**

 Citation was added

**Reviewer Comments:**

*The Hargreaves (* *Hargreaves, 1994b* *) equation demonstrated the nearest results to the classic PM (* *Droogers & Allen, 2002* *;* *Itenfisu et al., 2003* *). The Hargreaves method with substantially less data requirements, i.e., requiring only measured values of maximum and minimum temperatures and precipitation, was recommended for more general use and therefore is considered more appropriately applicable to data scarce regions and situations (* *Droogers & Allen, 2002* *). For the purposes of this paper, to maintain clarity, we refer to reference evapotranspiration calculated using the Penman-Monteith as “ET _0_” and calculations using the Hargreaves equation as “PET”. *Please refer to my above comment on the historical criticism of the uncalibrated Hargreaves model. The txt must provide more details in the point. 

**Author Response:**

 See point above

**Reviewer Comments:**

 There is a need for additional discussion of Aridity index (Vicente-Serrano et al. 2010 (Ref 6)). The original index was based on the use of the PET Thornthwaite model however recent a research has been criticized the use Thornthwaite model for assessing aridity severity (Tegos et al. 2023 (Ref 7)) and other PET models are recommended.  

**Author Response:**

 Indeed, some of the analyses results in the paper highlighted precisely the higher level of accuracy of Hargreaves respect to Thornthwaite method, among temperature-based methods. Herein, the choice of using Hargreaves rather ThornTwhaite for the calculation was substantially justified. Following this reviewer insight, we find some relevant merit to add in the usage note the following: *Aridity Index provides estimate meteorological droughts on annual temporal scale, while other methods (e.g. SPEI, PSDI) can be further implemented using these and Worldclim datasets to derive other more specific agrometeorological drought indicators at monthly and seasonal scale (Vicente-Serrano et al. 2010; Tegos et al. 2023).*

**References**

 1. Tabari H, Talaee P: Local Calibration of the Hargreaves and Priestley-Taylor Equations for Estimating Reference Evapotranspiration in Arid and Cold Climates of Iran Based on the Penman-Monteith Model. *Journal of Hydrologic Engineering. *2011; **16** (10): 837-845 | Publisher Full Text

 2. Haslinger K, Bartsch A: Creating long term gridded fields of reference evapotranspiration in Alpine terrain based on a re-calibrated Hargreaves method. 2015; | Publisher Full Text

 3. Tegos A, Malamos N, Efstratiadis A, et al.: Parametric Modelling of Potential Evapotranspiration: A Global Survey. *Water. *2017; **9** (10): | Publisher Full Text

 4. Tegos A, Malamos N, Koutsoyiannis D: A parsimonious regional parametric evapotranspiration model based on a simplification of the Penman–Monteith formula. *Journal of Hydrology. *2015; **524** 708-717 | Publisher Full Text

 5. McMahon T, Finlayson B, Peel M: Historical developments of models for estimating evaporation using standard meteorological data. *WIREs Water. *2016; **3** (6): 788-818 | Publisher Full Text

 6. Vicente-Serrano S, Beguería S, López-Moreno J: A Multiscalar Drought Index Sensitive to Global Warming: The Standardized Precipitation Evapotranspiration Index. *Journal of Climate. *2010; **23** (7):1696-1718 | Publisher Full Text

 7. Tegos A, Stefanidis S, Cody J, et al.: On the Sensitivity of Standardized-Precipitation-Evapotranspiration and Aridity Indexes Using Alternative Potential Evapotranspiration Models. *Hydrology. *2023; **10** (3): | Publisher Full Text

# Reviewer report: Reviewer response for version 1

**DOI:** 10.21956/openreseurope.19576.r43859

**Published:** 2024-09-27

**Authors:** Louise Crochemore, Camille Crapart

**Affiliations:** 1 Institute of Environmental Geosciences - CNRS, INRAE, IRD, Grenoble-INP, Universite Grenoble Alpes, Grenoble, France; 2 nstitute of Environmental Geosciences - CNRS, INRAE, IRD, Grenoble-INP, Université Grenoble Alpes, Grenoble, France

**Summary**

 Zomer et al. propose an open dataset of projected evapotranspiration (ET) and aridity index (AI) for the globe and at high resolution. The projected datasets are based on CMIP6 earth system models, across 4 emission scenarios and for the time period 2021-2040 and 2041-2060. This dataset builds upon the datasets and methodologies used in previously existing datasets of ET and AI for the historical period also produced by the authors.

 We have evaluated this work from a user perspective, and our comments mainly highlight clarifications needed to facilitate the use/understanding of the dataset and ensure that the provided information allows for its proper use. The work seems robust but addressing some of these points would strengthen this assessment.

**General comments**

- 22 CMIP6 models are mentioned, but only 14 can be found openly on the WorldClim projection datasets at the resolution of 30 arcsec. ( https://worldclim.org/data/cmip6/cmip6_clim30s.html). ACCESS-ESM1-5; CanESM5; CanESM5-CanOE; CNRM-CM6-1, CNRM-CM6-1-HR; CNRM-ESM2-A; GISS-E2-1-G; INM-CM4-8; MIRO-ES2L,MPI-ESM1-2-LR do not seem to appear. Conversely, the model EC-Earth3-Veg is available in Worldclim, and not used in this study. It would be useful for readers who want to replicate or use the dataset, for comparison for instance, to know (a) where the missing downscaled models were obtained from, and (b) why not all available models were used.
- The authors seem to use the terminologies of “potential evapotranspiration” and “reference evapotranspiration” interchangeably. Other datasets or articles have made a clear distinction between the two terms (Bellino et al. 2020; Xiang et al. 2020) and Allen et al. (1998) discourage the use of the term “potential evapotranspiration”. It seems paramount to clarify the definitions as early as possible in the note, to ease understanding and limit potential misuses of the dataset. Specific instances where the use of the terms can be confusing are listed in the detailed comments below.
- Given the amount of datasets, the note is at times hard to follow, and in particular the justification of the methods used and the validation (Methods and Technical Validation section). One outline that may be recommended would be to cover successively :
  - The comparison of the historical climate databases - currently : in Technical validation, there is first a comparison of WorldClim with CLIMWAT and, then only, the comparison with CRU. All these could be addressed at once and in a systematic manner. An early diagram or Table to summarize all historical datasets and their characteristics would be helpful in that regard.
  - The choice of the evapotranspiration equation - currently : in Methods, please see minor suggestions in the comments below
  - The validation of the ESMs of CMIP6 for the future indices proposed in this work - currently : this aspect is missing and would be valuable.
- The future AI is calculated for the periods 2021-2041 and 2041-2061 that are available in the CMIP6 downscaled database of Worldclim. Could the authors comment on why they do not calculate the indices and produce the maps for the horizons 2061-2081 and 2081-2100 as well?

**Detailed comments**

**Section « Abstract »**

- The database is named three times with three different names/formatting (“Future Global Aridity Index and PET Database”, “Future_Global_AI_PET Database”, Future_Global_AI_PET Database). A more consistent naming is needed.

**Section « Introduction »**

 §1

- « *Estimates* *of reference evapotranspiration (ET0) have been widely* *used across diverse scientific fields and practical domains* » : The reference evapotranspiration should be defined in this paragraph.
- « *with PET measurements* *and related indices playing a crucial role in agricultural* *and natural resource management* » : This sentence might apply for simulated PET as well. Please consider expanding on the word « measurements », either including simulated PET, or detailing the types of PET measurements, and why measurements specifically. Additionally, it would be helpful to give examples of or define the « related indices » the authors refer to.
- « *these metrics, along with their* *derived indices, assume a pivotal role* » We would suggest replacing the word « metrics » with a more specific word as it is unclear whether it is reference ET, PET measurements, or PET in general. 
- « *such measurements* » : here as well we would suggest being more specific to avoid confusion, and considering whether « measurements » is what the authors mean.

§2

- Introducing a sense of the time windows and time horizons in this paragraph might be helpful to avoid misconceptions of the use of the aridity index. « current conditions » can be seen as the current day, week, month, decade or decades. Similarly, « anticipated changes » are here likely over the coming decades. When mentioning the « operational decision-making processes” as well, irrigation management, crop cultivation practices and the anticipation of drought and flood likely require different time horizons as these decisions will be taken at different frequencies and anticipation times.

§3

- “ *Due to a continuing lack of high resolution downscaled CMIP6 future projection data for several climate variables needed to parameterize a full implementation of the Penman-Monteith equation, and a desire to maintain continuity to use the WorldClim datase* *ts (reportedly the most widely used climatic datasets for environmental, biogeographical or ecological analysis (Bobrowski et al., 2021)), a similar approach and methodology as used to develop the first version of the Global-AI_PET, i.e., which relied on t* *he Hargreaves equation for calculating PET, was applied to the CMIP6 ESM data.*” Three datasets are introduced in this sentence. If CMIP6 projection data are well introduced, WorldClim and Global-AI_PET might need references, links (provided in the following sentence for WorldClim) and indications of whether they cover the historical and/or future periods. Given the amount of information added, consider splitting the sentence in two.
- “ *Using bias-corrected downscaled data (30 arc seconds resolution) provided by WorldClim.org,*” Here as well, we would suggest elaborating on the word “data”, e.g. which variables, which periods.
- Here is the first occurrence in the main text of the database name “Future_Global_AI_PET”. It would be suitable to remind the full database name and make it clear that this dataset is the one presented here as the name of the Data Note does not make it clear.

**Section « Methods »**

**Sub-section « Earlier efforts and previous datasets** **»**

- This section clearly sets the context for this new dataset, showing that all previous efforts were on historical datasets. As such, it would be preferable to place it before §3 of the introduction (related to the first comment on §3).

**Sub-section « Estimating potential evapotranspiration** **»**

- *“FAO-56 defined PET as the ET of a reference crop (ET0) under optimal conditions.”* This statement seems inaccurate as Allen et al. (1998) in FAO-56 discourage the use of the term “potential evapotranspiration”.
- *“independently of specific crop characteristics, crop development stages, and management practices*” : Should water availability appear in this list?
- “ *As such, additional crop-specific coefficients (Kc) can be employed to derive ET estimates for specific crops (ETc), which can, in turn, be deduced from ET0*.” : We would suggest rephrasing this sentence into something along the lines of “As such, additional crop-specific coefficients (Kc) and ET0 can, in turn, be employed to derive ET estimates for specific crops (ETc).”
- “ *Centre Commun de Recherche of the European Economic Community*” : Consider using the English name : “European Commission’s Joint Research Centre”
- “ *A study of ET0 equations using lysimeter ET and synoptic climatic data by Centre Commun de Recherche of the European Economic Community (Choisnel et al., 1992) in 1992 of ET0 equations compared twelve equations using the classical Penman as the reference m* *ethod (Hargreaves, 1994b)*.” : Consider reformulating, for instance by removing “in 1992 of ET0 equations”.
- “ *For the purposes of this paper, to maintain clarity, we refer to reference evapotranspiration calculated using the Penman-Monteith as “ET0” and calculations using the Hargreaves equation as “PET*”. : This notation is contradictory with the following paragraph where “five different temperature-based methods of calculating PET were tested” including Hargreaves, Penman-Monteith, and others. See also main comment on the two notations.
- “ET0 values were calculated using the more complex Penman–Monteith model” : Please specify “more complex” or ideally give the Penman-Monteith equation you use. Additionally, check the spelling of “Penman-Monteith” as several spellings are found in the text (Penmen, Montieth). Lastly, the abbreviation “PM” is used in some instances but not systematically.

**Sub-section « Hargreaves equation** **»**

- “calculate ET0, as shown below: PET =” : This is another occurrence where the abbrevations used are confusing, especially with ET0 defined earlier as the Penman-Monteith reference evapotranspiration, and PET as the Hargreaves one.
- “The data to parametrize the equation were obtained from the WorldClim.org online data repository (Fick & Hijmans, 2017), which provides bias-corrected downscaled monthly values of minimum temperature, maximum temperature, and precipitation for 24 CMIP6 Earth System Models (ESMs), for each of four SSP: 126, 245, 370 and 585.” In the introduction, the authors mention 22 models. Which number is correct? (see also previous comment on the models not found in Worldclim).
- The reference to Wang et al. (2018) for extraterrestrial radiation is likely erroneous. Please check.
- “PET and AI were estimated for two historical periods, WorldClim 1.4 (1960–1990) and WorldClim 2.1 (1970–2000), representing on average a decades’ change, by applying the Hargreaves methodology described above.” From what we understand in the “technical validation” section, the data from Worldclim 1.4 is used to calculate PET and AI with the Hargreaves equation for the period 1960-1990, and the data from Worldclim 2.1 is used to calculate ET0 and AI with Penman-Monteith equation for the period 1970-2000. Is that correct and is the Hargreaves equation also applied on the data from Worldclim 2.1?

**Sub-section « Multi-model ensembles** **»**

- The idea of creating 3 different means is interesting. The choice of the 5 “outliers” seems clear based on Figure 3, that plots mean annual temperature against mean annual precipitation for the period 2021-2040 in the 4 SSP. However, it is not as visible for the period 2041-2060, where other models could be identified as “outliers” (Figure 4). Was the choice made primarily based on the comparison for the 2021-2040 period? Would there be another way to justify the choice of these 5 models as “high risk”?
- Level of agreement and coefficient of variation : This metric is used to compare the agreement of the models within each of the 3 multimodel means. Table 4 is only briefly commented in  “Agreement was fairly high for the basic parameters of annual average temperature and annual average precipitation, somewhat less for annual averaged PET and AI, slightly increasing in the 2041–2060 time period.” It would be interesting to explain what “fairly high” means. All the CoV are under 5% (except for the aridity index). It could be worth noting that the “all” multimodel ensemble does have higher CoV than the other 2, but rather low as well. Also, it makes sense that the “consensus” and “high risk” ensembles have lower CoV because they include fewer models, at least for the period 2021-2040 for which they were chosen. In addition, there is no difference between CoV aridity index for “all” and “high risk” for the period 2041-2060, probably because the choice of these 5 “high risk” models is less relevant for this period?
- Concerning the regional differences: maybe some examples?
- Consider identifying the models constituting the High Risk and the Majority consensus ensembles on Figures 3 and 4.
- “for the each of the two time period” : replace with “for each of the two time periods”

**Section « Technical validation »**

- The discussion over the previously published dataset is already available in Zomer 2022 **.** This analysis is presented again, here. For the sake of clarity, consider whether it is necessary to present these results again or if simply referring to this work could be enough.
- In addition, using the CLIMWAT and CRU data to compute PM-ET0, and then comparing with the PM-ET0 from Worldclim, obviously leads to better results than using Hargreaves. We know from the start that the Hargreaves equation will be chosen, because there is not enough variables in the downscaled CMIP6 models to use the Penman-Monteith equation. The point of using the Hargreaves equation instead of Penman-Monteith has been treated in the Methods section. It is hard to understand why so many steps of technical validation are necessary to prove that it is possible to use this equation.
- The criticism on the caveats of the evapotranspiration calculation are interesting, but they apply to both Penman-Monteith and Hargreaves, and could appear immediately after the comparison of the two equations with observation data.
- The presentation of the CRU_TS dataset arrives late in the note. As mentioned previously, it would be helpful to have all datasets introduced in a section earlier in the text.
- Worldclim is validated against CLIMWAT. What is the period covered by CLIMWAT? (in Zomer 2022: “roughly” 1970-2000, but more precisely)? The CRU data is said to be averaged for the period 1970-2000. It would make sense that all databases use the same time period. In that regard, it would also make sense to compare the Aridity index calculated with Hargreaves on the Worldclim 2.1 database, instead of using the Global-AI_PET_v1, that is calculated for the period 1960-1990.
- The number of CLIMWAT weather stations changes throughout this section (from 5000 to 4242 to 3842). Please explain when presenting CLIMWAT the different subsets used and for which purpose.
- “very fairly high” : needs reformulating.
- “However, the AI estimates based on the Global-AI_PET_v1 analysis, when compared to AI estimates based on parameters provided by the CLIMWAT weather station data (Table 5; Figure 9e), showed a high level of correspondence (r2 = 0.91), statistically the same but nominally slightly higher than that from the Global-AI_PET_v3 estimates (r2 = 0.90).” Does that mean that the bias on precipitation compensates for the bias in evapotranspiration?
- “statistically the same but nominally slightly higher than” : in all fairness, if they cannot be statistically distinguished, the fact that one is higher than the other is not significant and cannot be exploited.
- “the relatively (or moderately) high accuracy (r2 = 0.72) attained by the Hargreaves implementation of the algorithm is considered sufficient for use within many modeling and other efforts”. What is the exact meaning of “is considered sufficient”? To which extent is there a choice given the available variables?
- “Results showed a moderate level of agreement (r2 = 0.67) between the ET0_CRU_TS and Hargreaves (Global_PET_v1) data, with a significantly higher level of agreement, as expected, for the Penman-Monteith (Global_ET0_v1) results.” What is the r2 for the second comparison? Also, Global_ET0_v1 has not been presented in “earlier efforts and previous datasets” and appears directly in Table 5.
- “mission critical purposes” : could you clarify what you mean by this, by reformulating or maybe giving examples?
- “the both” : remove “the”

**Tables**

- Table 2 : We suggest mentioning the reference in the caption. The caption currently states “between observed and predicted estimates”, but if our understanding is correct it is the Penman-Monteith model applied to observations.
- Table 5: Check for typos (“regression”, “elevation”). This table is helpful as it recaps (almost all) datasets used here. As such, it would be helpful to show the list of datasets used in the study earlier on in the text. See also the comment on the comparison diagram.

**Figures**

- Better resolution in Figures may be recommended, particularly for Figures 3 and 4 which can be hard to read.
- Figure 2 : The label of the Thornthwaite method overlaps with the southern part of Africa. A reference to the previous report ( https://www.iwmi.cgiar.org/Publications/IWMI_Research_Reports/PDF/pub101/RR101.pdf) may be needed.
- Figure 7 : This figure could be further commented on. For instance: Most of the “high risk’ averages are wetter than the other averages in the period 2021-2041. The “consensus” and “all” means have the same mean global aridity index, except for the SSP 585 which is much drier in the “consensus” than in “all”. It seems that there should be 6 points for each SSP, but, for instance, only 3 points correspond to SSP 585. Similarly, the white triangles for the 2041-2060 High Risk ensemble appear in the legend but do not appear in the figure.
- Figure 10 : The caption states “Hargreaves PET” but the figure labels are “ET0”.

Are sufficient details of methods and materials provided to allow replication by others?

Partly

Is the rationale for creating the dataset(s) clearly described?

Yes

Are the datasets clearly presented in a useable and accessible format?

Yes

Are the protocols appropriate and is the work technically sound?

Partly

Reviewer Expertise:

For the two reviewers involved : ET modelling ; Assessment of Future Climate ; Global Hydrological Modelling

We confirm that we have read this submission and believe that we have an appropriate level of expertise to confirm that it is of an acceptable scientific standard, however we have significant reservations, as outlined above.

## References

[1] : Similarity and difference of potential evapotranspiration and reference crop evapotranspiration – a review. *Agricultural Water Management* .2020;232: 10.1016/j.agwat.2020.106043 10.1016/j.agwat.2020.106043

[2] : Daily reference and potential evapotranspiration, and supporting meteorological data from the North American Regional Reanalysis, solar insolation data from the GOES satellite, and blue-sky albedo data from the MODIS satellite, Florida, 2019. *U.S. Geological Survey data release,* .2020; 10.5066/P9Y8VTQC 10.5066/P9Y8VTQC

# Response

**Published:** 2025-01-29

**Authors:** Robert Zomer

**Affiliations:** IAFES, Foundation Euro-Mediterranean Center for Climate Change, Sassari, Sardinia, Italy

**Response to Reviewer #2** **Camille Crapart**, Institute of Environmental Geosciences - CNRS, INRAE, IRD, Grenoble-INP, Universite Grenoble Alpes, Grenoble, France **Louise Crochemore, **Institute of Environmental Geosciences - CNRS, INRAE, IRD, Grenoble-INP, Université Grenoble Alpes, Grenoble, France   The authors thank the reviewers for their effort in reviewing this manuscript, and their thorough and  detailed comments.  The authors have strived to address all the points as best we can, as we understand them, and believe it has improved the manuscript and its readability.  Again we a heartened by the evalution of the reviewers that the work “seems robust”.  

**General comments**

- 22 CMIP6 models are mentioned, but only 14 can be found openly on the WorldClim projection datasets at the resolution of 30 arcsec. ( https://worldclim.org/data/cmip6/cmip6_clim30s.html). ACCESS-ESM1-5; CanESM5; CanESM5-CanOE; CNRM-CM6-1, CNRM-CM6-1-HR; CNRM-ESM2-A; GISS-E2-1-G; INM-CM4-8; MIRO-ES2L,MPI-ESM1-2-LR do not seem to appear. Conversely, the model EC-Earth3-Veg is available in Worldclim, and not used in this study. It would be useful for readers who want to replicate or use the dataset, for comparison for instance, to know (a) where the missing downscaled models were obtained from, and (b) why not all available models were used.

**Response: **You are completely correct in that at the time of your review there were only 14 ESM on the WorldClim website.  However, when we first downloaded these datasets (in 2022) there were in fact 25 ESM available online.  We contacted Dr. Robert Hijmans at UC Davis, who is responsible for the WorldClim datasets, to enquire what happened to the other datasets and if there were any issues associated with them not being available that we should know about. He assured us that all the datasets were still available online and provided us with an updated link where all of the 25 datasets are archived.  He also assured us that there were no issues, however he had made a selection of the higher resolution (in original processing) ESM to reduce bandwidth and load on his site.  We have updated the link to the datasets in the text.   As it turns out, there is a link to the page with all the datasets on the very bottom of the page we referenced.  Where it says: “For some GCMs there are variations available here.”   Here is the updated link.  We have revised this in the text. https://worldclim.org/data/cmip6_all/cmip6_clim30s.html   We found spatial artifacts (visible cross-hatching across large areas of land) in the two EC-Earth3-Veg ESMs, which we deemed made them unusable, so we dropped them from the database and also from inclusion in any ensembles.  This issue was already there in the original files.  We have informed WorldClim regarding this issue.  

- The authors seem to use the terminologies of “potential evapotranspiration” and “reference evapotranspiration” interchangeably. Other datasets or articles have made a clear distinction between the two terms (Bellino et al. 2020; Xiang et al. 2020) and Allen et al. (1998) ****discourage the use of the term “potential evapotranspiration”. It seems paramount to clarify the definitions as early as possible in the note, to ease understanding and limit potential misuses of the dataset. Specific instances where the use of the terms can be confusing are listed in the detailed comments below.

**Response:**  We have gone through the paper and now all references to Penman-Monteith Reference ET is referred to as “ET _0_”, and all references to other methods, primarily Hargreaves as implemented in this paper, are referred to as “PET  

- Given the amount of datasets, the note is at times hard to follow, and in particular the justification of the methods used and the validation (Methods and Technical Validation section). One outline that may be recommended would be to cover successively :
  - The comparison of the historical climate databases - currently : in Technical validation, there is first a comparison of WorldClim with CLIMWAT and, then only, the comparison with CRU. All these could be addressed at once and in a systematic manner. An early diagram or Table to summarize all historical datasets and their characteristics would be helpful in that regard.

**Response:**  We have slightly revised the order, and left most of the comparison in the Technical Validation section, since this is the purpose of presenting this particular data. Table 1 summarizes all historical and other datasets together at once, as suggested.

-
  - The choice of the evapotranspiration equation - currently : in Methods, please see minor suggestions in the comments below

**Response: **We have responded to these below:

-
  - The validation of the ESMs of CMIP6 for the future indices proposed in this work - currently : this aspect is missing and would be valuable.

**Response: **We agree that a more rigorous evaluation of the ESM’s themselves would be valuable. beyond the historical comparisons and measures of uncertainty (ensemble coefficients of variability) we provide. However, as we provide a full complement of ESMs (n = 22), and there is significant regional differentiation and a range of scale and thematic applicability amongst the ESM, we provide this as a global public good and leave the choice of which model and how to use it up to the user.  There is a large body of literature available on this topic to provide guidance.  

- The future AI is calculated for the periods 2021-2041 and 2041-2061 that are available in the CMIP6 downscaled database of Worldclim. Could the authors comment on why they do not calculate the indices and produce the maps for the horizons 2061-2081 and 2081-2100 as well?

**Response: ** Two considerations went into our decision to limit our analyzes to the two time periods 2021-2041 and 2041-2061: First, computing constraints and data storage capacity.  The current work represents nearly a year, or more of processing time already.  And second, ESM uncertainty increases as the time period is extended into the future, making the results less operationally useful or practical for policy purposes.      

**Detailed comments**

**Section « Abstract »**

- The database is named three times with three different names/formatting (“Future Global Aridity Index and PET Database”, “Future_Global_AI_PET Database”, Future_Global_AI_PET Database). A more consistent naming is needed.

**Response: **All instances in the manuscript are now revised to: Future_Global_AI_PET Database

**Section « Introduction »**

 §1

- « *Estimates of reference evapotranspiration (ET0) have been widely used across diverse scientific fields and practical domains* » : The reference evapotranspiration should be defined in this paragraph.

**Response: **We have added the following sentence to the paragraph.  Reference ET is more specifically defined in the Methods section: “While potential evapotranspiration (PET) reflects maximum possible water loss across varied vegetation types, useful in broader ecosystem and hydrological studies, reference evapotranspiration (ET₀) estimates the maximum possible water loss from a well-watered reference crop, and as such, is particularly useful for agriculture and irrigation management..”

- « *with PET measurements and related indices playing a crucial role in agricultural and natural resource management* » : This sentence might apply for simulated PET as well. Please consider expanding on the word « measurements », either including simulated PET, or detailing the types of PET measurements, and why measurements specifically. Additionally, it would be helpful to give examples of or define the « related indices » the authors refer to.

**Response: **We have changed the following sentences: “Estimates of reference evapotranspiration (ET _0_) have been widely used across diverse scientific fields and practical domains ( Hargreaves, 1994a; Zomer *et al.*, 2022), with PET and various indices, playing a crucial role in agricultural and natural resource management” “As an example, indices relying on PET estimates include the Standardized Precipitation Evapotranspiration Index (SPEI), Palmer Drought Severity Index (PDSI), Crop Water Stress Index (CWSI), Water Deficit Index (WDI), Evaporative Stress Index (ESI), and the Aridity Index (AI), Indices like the SPEI, AI, PDSI, CWSI, WDI, and ESI incorporate PET to understand water availability, climate impacts, and drought.”

- « *these metrics, along with their derived indices, assume a pivotal role* » We would suggest replacing the word « metrics » with a more specific word as it is unclear whether it is reference ET, PET measurements, or PET in general. 

**Response: **Actually, the intention is to not be specific, but refer to PET estimates as a class in general (that is, as we see it ET _0_ is a subset of PET, so when we refer to PET, it can be inclusive of ET _0_).  We have changed the sentence to: “In an era of rapid environmental and climatic transformations, PET estimates, along with their related indices, assume a pivotal role as direct and critical reference measures.

- « *such measurements* » : here as well we would suggest being more specific to avoid confusion, and considering whether « measurements » is what the authors mean.

**Response: **Changed to “estimates” .

 §2

- Introducing a sense of the time windows and time horizons in this paragraph might be helpful to avoid misconceptions of the use of the aridity index. « current conditions » can be seen as the current day, week, month, decade or decades. Similarly, « anticipated changes » are here likely over the coming decades. When mentioning the « operational decision-making processes” as well, irrigation management, crop cultivation practices and the anticipation of drought and flood likely require different time horizons as these decisions will be taken at different frequencies and anticipation times.

**Response: **To facilitate understanding of time horizons we have changed the sentence to: “The AI serves as proxy for a comprehensive evaluation of a set of hydro-climatological and hydro-ecological variables to determine historical, recent conditions and projected changes in hydrological cycles at specified future times and time scales.” §3

- “ *Due to a continuing lack* *of high resolution downscaled CMIP6 future projection data for several climate variables needed to parameterize a full implementation of the Penman-Monteith equation, and a desire to maintain continuity to use the WorldClim datasets (reportedly the most widely used climatic datasets for environmental, biogeographical or ecological analysis (Bobrowski et al., 2021)), a similar approach and methodology as used to develop the first version of the Global-AI_PET, i.e., which relied on the Hargreaves equation for calculating PET, was applied to the CMIP6 ESM data.*” Three datasets are introduced in this sentence. If CMIP6 projection data are well introduced, WorldClim and Global-AI_PET might need references, links (provided in the following sentence for WorldClim) and indications of whether they cover the historical and/or future periods. Given the amount of information added, consider splitting the sentence in two.

**Response: **We have revised the sentence as per below: “A set of future projections  of global PET and AI ( Figure 1) were produced to construct the Global-Future_AI_PET Database (Zomer and Trabucco, 2024), based on 22 Coupled Inter-Comparison Modeling Project – Phase 6 (CMIP6: (Eyring et al., 2016)) Earth System Models (ESM). Due to a continuing lack of high resolution downscaled CMIP6 future projection data for several climate variables needed to parameterize a full implementation of the Penman-Monteith equation, and a desire to maintain continuity with the previously used WorldClim (Fick and Hijmans, 2017) datasets (reportedly among the most widely used climatic datasets for environmental, biogeographical or ecological analysis ( Bobrowski *et al.*, 2021)), Hargreaves equation for calculating PET, was applied to the CMIP6 ESM data. This maintains a similar approach and methodology as used to develop the Global-AI_PET Database v1 (Trabucco and Zomer, 2008).

- “ *Using bias-corrected downscaled data (30 arc seconds resolution) provided by WorldClim.org,*” Here as well, we would suggest elaborating on the word “data”, e.g. which variables, which periods.

**Response: **We have revised the sentence as per below: This maintains a similar approach and methodology as used to develop the Global-AI_PET Database v1 (Trabucco and Zomer, 2008). Using bias-corrected downscaled climatic parameters (i.e., minimum / maximum temperature, and precipitation), derived from interpolation of weather station data (30 arc seconds resolution), and provided by WorldClim.org, each ESM model projection was evaluated across four emission scenarios, or *Shared Socio-Economic Pathways* (SSP 126; 245; 370; 585).

- Here is the first occurrence in the main text of the database name “Future_Global_AI_PET”. It would be suitable to remind the full database name and make it clear that this dataset is the one presented here as the name of the Data Note does not make it clear.

**Response: **Revised to: “The Future_Global_AI_PET Database (Zomer & Trabucco, 2024) described in this paper is archived and available online from the Science Data Bank (ScienceDB) at: https://doi.org/10.57760/sciencedb.nbsdc.00086.”

**Section « Methods »**

**Sub-section « Earlier efforts and previous datasets »**

- This section clearly sets the context for this new dataset, showing that all previous efforts were on historical datasets. As such, it would be preferable to place it before §3 of the introduction (related to the first comment on §3).

**Response: **As suggested, we have moved this paragraph and placed it before §3.  

**Sub-section « Estimating potential evapotranspiration »**

- *“FAO-56 defined PET as the ET of a reference crop (ET0) under optimal conditions.”* This statement seems inaccurate as Allen et al. (1998) in FAO-56 discourage the use of the term “potential evapotranspiration”.

**Response: ** We have replaced this sentence with the following: “FAO-56 defines and provide guidance on calculating reference evapotranspiration (ET₀), that is, the evapotranspiration (ET) of a reference crop, rather than defining potential evapotranspiration (PET) directly. FAO-56 presents a standard method to estimate ET₀, which is widely accepted as a reference standard, and often closely related to PET in practice. In FAO-56, the ET₀ is calculated using the Penman-Monteith equation, which takes into account factors like temperature, relative humidity, wind speed, and solar radiation. While ET₀ is a measure of the evapotranspiration rate from a reference crop under optimal conditions, PET generally represents the water demand of vegetation if water is freely available. Though not synonymous, ET₀ from FAO-56 is widely used as an estimate for PET, particularly in agricultural and environmental applications.”  

- *“independently of specific crop characteristics, crop development stages, and management practices*” : Should water availability appear in this list?

**Response: **Changed to:  “independent of specific crop characteristics, crop development stages, management practices, or water availability”

- “ *As such, additional crop-specific coefficients (Kc) can be employed to derive ET estimates for specific crops (ETc), which can, in turn, be deduced from ET0*.” : We would suggest rephrasing this sentence into something along the lines of “As such, additional crop-specific coefficients (Kc) and ET0 can, in turn, be employed to derive ET estimates for specific crops (ETc).”

**Response: **Changed to: “As such, additional crop-specific coefficients (Kc) and ET _0_ can, in turn, be employed to derive ET estimates for specific crops (ETc).”

- “ *Centre Commun de Recherche of the European Economic Community*” : Consider using the English name : “European Commission’s Joint Research Centre”

**Response: **Changed to:   “European Commission’s Joint Research Centre”

- “ *A study of ET0 equations using lysimeter ET and synoptic climatic data by Centre Commun de Recherche of the European Economic Community (Choisnel et al., 1992) in 1992 of ET0 equations compared twelve equations using the classical Penman as the reference method (Hargreaves, 1994b)*.” : Consider reformulating, for instance by removing “in 1992 of ET0 equations”.

**Response: **Changed to: “A study of ET _0_ equations using lysimeter ET and synoptic climatic data by European Commission’s Joint Research Centre ( Choisnel *et al.*, 1992) compared twelve equations using Penman-Monteith (Allen et al., 1998)as the reference standard.”

- “ *For the purposes of this paper, to maintain clarity, we refer to reference evapotranspiration calculated using the Penman-Monteith as “ET0” and calculations using the Hargreaves equation as “PET*”. : This notation is contradictory with the following paragraph where “five different temperature-based methods of calculating PET were tested” including Hargreaves, Penman-Monteith, and others. See also main comment on the two notations.

**Response: **Now change to: “four different temperature-based methods of calculating PET  were compared with the Penman-Montieth method for calculating ET _0_, and tested to determine which equation”

- “ET0 values were calculated using the more complex Penman–Monteith model” : Please specify “more complex” or ideally give the Penman-Monteith equation you use. Additionally, check the spelling of “Penman-Monteith” as several spellings are found in the text (Penmen, Montieth). Lastly, the abbreviation “PM” is used in some instances but not systematically.

**Response: **Penman-Monteith equation is now given in the paper.  

**Sub-section « Hargreaves equation »**

- “calculate ET0, as shown below: PET =” : This is another occurrence where the abbrevations used are confusing, especially with ET0 defined earlier as the Penman-Monteith reference evapotranspiration, and PET as the Hargreaves one.

**Response: **This was a mistake.. Changed to:   “to calculate PET, as shown below:”

- “The data to parametrize the equation were obtained from the WorldClim.org online data repository (Fick & Hijmans, 2017), which provides bias-corrected downscaled monthly values of minimum temperature, maximum temperature, and precipitation for 24 CMIP6 Earth System Models (ESMs), for each of four SSP: 126, 245, 370 and 585.” In the introduction, the authors mention 22 models. Which number is correct? (see also previous comment on the models not found in Worldclim).

**Response: **Corrected to:  22

- The reference to Wang et al. (2018) for extraterrestrial radiation is likely erroneous. Please check.

**Response: **No… not erroneous.  This paper has an extensive discussion of extraterrestrial radiation.

- “PET and AI were estimated for two historical periods, WorldClim 1.4 (1960–1990) and WorldClim 2.1 (1970–2000), representing on average a decades’ change, by applying the Hargreaves methodology described above.” From what we understand in the “technical validation” section, the data from Worldclim 1.4 is used to calculate PET and AI with the Hargreaves equation for the period 1960-1990, and the data from Worldclim 2.1 is used to calculate ET0 and AI with Penman-Monteith equation for the period 1970-2000. Is that correct and is the Hargreaves equation also applied on the data from Worldclim 2.1?

**Response: **We calculate PET and AI using the Hargreaves equation for both WorldClim 1.4 (1960-1990) and WorldClim 2.1 (1970-2000). Both these datasets are included in the database under Historical Datasets. In addition, WorldClim 2.1 was used to calculate ET0 and AI with Penman-Monteith, as described in an earlier paper, but this is not included in the Future_Global_AI_PET Database. We refer to it only for comparative purposes.

**Sub-section « Multi-model ensembles »**

- The idea of creating 3 different means is interesting. The choice of the 5 “outliers” seems clear based on Figure 3, that plots mean annual temperature against mean annual precipitation for the period 2021-2040 in the 4 SSP. However, it is not as visible for the period 2041-2060, where other models could be identified as “outliers” (Figure 4). Was the choice made primarily based on the comparison for the 2021-2040 period? Would there be another way to justify the choice of these 5 models as “high risk”?

**Response: **The reviewers point out an important observation regarding the selection of the 5 high risk models. These were selected primarily based on the comparison for the 2021-2040 period, with some consideration of the 2041-2060 period.  However, choosing different set of models for different periods could also highlight some inconsistencies across the trends over the future time periods. We recognized the shortcomings of making optimal choice. However, we provide these only as examples, but the database contains all 22 models, allowing users to construct their own ensemble combinations.

- Level of agreement and coefficient of variation : This metric is used to compare the agreement of the models within each of the 3 multimodel means. Table 4 is only briefly commented in  “Agreement was fairly high for the basic parameters of annual average temperature and annual average precipitation, somewhat less for annual averaged PET and AI, slightly increasing in the 2041–2060 time period.” It would be interesting to explain what “fairly high” means. All the CoV are under 5% (except for the aridity index). It could be worth noting that the “all” multimodel ensemble does have higher CoV than the other 2, but rather low as well. Also, it makes sense that the “consensus” and “high risk” ensembles have lower CoV because they include fewer models, at least for the period 2021-2040 for which they were chosen. In addition, there is no difference between CoV aridity index for “all” and “high risk” for the period 2041-2060, probably because the choice of these 5 “high risk” models is less relevant for this period?

**Response: **Change sentence to:  “Agreement was fairly high for the basic parameters of annual average temperature and annual average precipitation with all values below 5%, indicating very low variability relative to the mean, suggesting strong consistency or agreement. Somewhat less for annual averaged PET and AI, which is slightly increased in the 2041–2060 time period.”   We are not entirely sure why there is no difference in the 2041-2060 period, but we don’t think it is because the selected 5 are less relevant.

- Concerning the regional differences: maybe some examples?

**Response: **Since this is a data note, and not a research article, we really prefer not to present too many results, other than as required for the user of the dataset. Even though we have indeed done all this analysis, we leave this to the user to explore.  There really is just too much of it to present, and CoV is not arguably that prominent an issue.

- Consider identifying the models constituting the High Risk and the Majority consensus ensembles on Figures 3 and 4.

**Response: **Yes, we have considered this, but difficult to differentiate as is with the number of overlapping models.

- “for the each of the two time period” : replace with “for each of the two time periods”

**Response: **Done.

**Section « Technical validation »**

- The discussion over the previously published dataset is already available in ****Zomer 2022 **.**This analysis is presented again, here. For the sake of clarity, consider whether it is necessary to present these results again or if simply referring to this work could be enough.

**Response: **It is the authors opinion that for the sake of clarity it is still valuable to have this discussion here, as we are referring to a dataset that is used as a comparison in the technical validation.

- In addition, using the CLIMWAT and CRU data to compute PM-ET0, and then comparing with the PM-ET0 from Worldclim, obviously leads to better results than using Hargreaves. We know from the start that the Hargreaves equation will be chosen, because there is not enough variables in the downscaled CMIP6 models to use the Penman-Monteith equation. The point of using the Hargreaves equation instead of Penman-Monteith has been treated in the Methods section. It is hard to understand why so many steps of technical validation are necessary to prove that it is possible to use this equation.

**Response: **Yes, thank you for your advice and support on this.  We agree with your assessment. However, due to skepticism by a previous reviewer of the Hargreaves methodology, we added discussion on this topic for those that are skeptical or unaware of its wide use and utility. However, we agree that this is redundant and muddled the text with too many datasets and regressions. We have streamlined this now, taken out much of the ancillary data on the datasets (elev, temp, etc..) and revised Table 5 and both Figure 9 and 10.

- The criticism on the caveats of the evapotranspiration calculation are interesting, but they apply to both Penman-Monteith and Hargreaves, and could appear immediately after the comparison of the two equations with observation data.

**Response: **The authors agree that this discussion is more relevant to the Technical Validation section.

- The presentation of the CRU_TS dataset arrives late in the note. As mentioned previously, it would be helpful to have all datasets introduced in a section earlier in the text.

**Response: **The CRU_TS dataset is only used in the technical validation section, as such we introduce it here. Basically, we introduce the datasets used for comparison in sequence, corresponding to their relative figures.

- Worldclim is validated against CLIMWAT. What is the period covered by CLIMWAT? (in Zomer 2022: “roughly” 1970-2000, but more precisely)? The CRU data is said to be averaged for the period 1970-2000. It would make sense that all databases use the same time period. In that regard, it would also make sense to compare the Aridity index calculated with Hargreaves on the Worldclim 2.1 database, instead of using the Global-AI_PET_v1, that is calculated for the period 1960-1990.

**Response: **We have now emphasized the 1970-2000 dataset for comparison and cover roughly the same period as the CLIMWAT and the CRU_TS, i.e. 1970-2000. We continue to include the WC_1.4 data (1960-1990), since this is both included in the Future Global-AI_PET Database and because it has previously been downloaded and used many thousands of times, as a reference to those users. In regards the “roughly” 1970-2000 time period, from the CLIMWAT website:  In compiling the data, an effort was made to cover the period 1971 - 2000, but when data for this period were not available, any recent series that ends after 1975 and that has at least 15 years of data have been included. Some of the series are "broken", but they nevertheless have at least 15 years of data (e.g. 1961-70 and 1992-2000).”  So… “roughly” 1970-2000.

- The number of CLIMWAT weather stations changes throughout this section (from 5000 to 4242 to 3842). Please explain when presenting CLIMWAT the different subsets used and for which purpose.

**Response: **There are 5000 stations reported in the full CLIMWAT dataset. We were able to locate and use 4242 stations for the spreadsheet analysis (some stations didn’t seem to correspond to the ancillary data, e.g. the DEM). When we extracted the values from the geospatial datasets, there were various no_data values or other anomalies that we excluded. We then created a subset of available data points common among all datasets, which came out to 3842 stations.

- “very fairly high” : needs reformulating.

**Response: **Changed to “fairly high”

- “However, the AI estimates based on the Global-AI_PET_v1 analysis, when compared to AI estimates based on parameters provided by the CLIMWAT weather station data (Table 5; Figure 9e), showed a high level of correspondence (r2 = 0.91), statistically the same but nominally slightly higher than that from the Global-AI_PET_v3 estimates (r2 = 0.90).” Does that mean that the bias on precipitation compensates for the bias in evapotranspiration?

**Response: **This could perhaps be the case, however, we don’t really speculate on this, as again, this is a data note primarily intended to describe the data, and not really interpret results. But short answer, we are not really sure if this is the case, or not.

- “statistically the same but nominally slightly higher than” : in all fairness, if they cannot be statistically distinguished, the fact that one is higher than the other is not significant and cannot be exploited.

**Response: **Changed to: “statistically the same as that from”

- “the relatively (or moderately) high accuracy (r2 = 0.72) attained by the Hargreaves implementation of the algorithm is considered sufficient for use within many modeling and other efforts”. What is the exact meaning of “is considered sufficient”? To which extent is there a choice given the available variables?

**Response: **We have change this sentence to: “… maybe considered sufficient for use within many modeling and other efforts, however, with caveats in light of the variability associated with both the input data and accuracy of the estimation algorithm when applied globally.

- “Results showed a moderate level of agreement (r2 = 0.67) between the ET0_CRU_TS and Hargreaves (Global_PET_v1) data, with a significantly higher level of agreement, as expected, for the Penman-Monteith (Global_ET0_v1) results.” What is the r2 for the second comparison? Also, Global_ET0_v1 has not been presented in “earlier efforts and previous datasets” and appears directly in Table 5.

**Response: **This sentence has been revised to report 1970-2000 results: “Results showed a moderate level of agreement (r ^2^ = 0.68) between the ET _0__CRU_TS and Hargreaves (PET_WC_2.1) data, with a significantly higher level of agreement, as expected, for the Penman-Monteith(Global_ET _0__v3: r ^2^ = 0.89) results.”

- “mission critical purposes” : could you clarify what you mean by this, by reformulating or maybe giving examples?

**Response: **Removed “mission critical purposes”.

- “the both” : remove “the”

**Tables**

- Table 2 : We suggest mentioning the reference in the caption. The caption currently states “between observed and predicted estimates”, but if our understanding is correct it is the Penman-Monteith model applied to observations.

**Response: ** We have changed this to: “Results of the comparison are given as the mean difference (Mean Diff) between Penman_Monteith estimates based on observed weather station data and predicted estimates using the various different methods, and their standard deviations (Std Dev). Source: ( Trabucco *et al.*, 2008).”  

- Table 5: Check for typos (“regression”, “elevation”). This table is helpful as it recaps (almost all) datasets used here. As such, it would be helpful to show the list of datasets used in the study earlier on in the text. See also the comment on the comparison diagram.

**Response: ** Thanks… corrections made. **Figures**

- Better resolution in Figures may be recommended, particularly for Figures 3 and 4 which can be hard to read.

**Response: ** We have attempted to improve the figures 3 and 4, there are still some issues with overlap that can’t be helped. We believe that the overall pattern of the distribution is well presented however.

- Figure 2 : The label of the Thornthwaite method overlaps with the southern part of Africa. A reference to the previous report ( https://www.iwmi.cgiar.org/Publications/IWMI_Research_Reports/PDF/pub101/RR101.pdf) may be needed.

**Response: ** Sorry about the label… unfortunately, we just can’t seem to go back to find the original graphic files to fix that. Reference was given in the text, now added to caption.

- Figure 7 : This figure could be further commented on. For instance: Most of the “high risk’ averages are wetter than the other averages in the period 2021-2041. The “consensus” and “all” means have the same mean global aridity index, except for the SSP 585 which is much drier in the “consensus” than in “all”. It seems that there should be 6 points for each SSP, but, for instance, only 3 points correspond to SSP 585. Similarly, the white triangles for the 2041-2060 High Risk ensemble appear in the legend but do not appear in the figure.

**Response: ** There’s a paper that could be written on this figure, as it represents the main findings if one were to be analyzing the dataset. However, this is a “Data Note”, and not a research paper, as such, we present this as an overview of the distribution of the results for the user of the database, and do not attempt to interpret these results here.

- Figure 10 : The caption states “Hargreaves PET” but the figure labels are “ET0”.

**Response: ** Labeled revised to: “Validation and comparison of various results with the CRU_TS dataset.”

# Reviewer report: Reviewer response for version 1

**DOI:** 10.21956/openreseurope.19576.r42664

**Published:** 2024-08-05

**Authors:** Vassilis Aschonitis

**Affiliations:** 1 Hellenic Agricultural Organization, Soil and Water Resources Institute, Thessaloniki, Greece

The authors provided new high-resolution (30 arc-seconds) global raster datasets of average monthly and annual potential evapotransipation (PET) and aridity index (AI) for two historical (1960-1990; 1970-2000) and two future (2021-2040; 2041-2060) time periods for each of 22 CMIP6 Earth System Models across four emission scenarios (SSP: 126, 245, 370, 585). The database also includes three averaged multi-model ensembles produced for each of the four emission scenarios.

 The work is robust. Some minor comments are the following:

 1. The regressions in Fig.9 and 10 are based on y=ax  instead of y=ax+b. The R2 of the two regression lines are different with the first one providing artificially higher R2. The calculation of R2 for a linear regression with intercept forced to 0 is theoretically problematic and should be avoided (Eisenhauer, 2003). Another reason for not using linear regression with 0 intercept in 1:1 plots is that the regression line neglects information associated to the performance of a model in the low values.

 2. For the PET methods description, you give only the equations of Hargreaves but not the equations of the other four methods used in this study.

 3. Just for your consideration, there are newer modifications of Hargreaves and Thornthwaite methods based on ASCE Penman-Monteith method. For the original Hargreaves method, the study of Aschonitis et al. (2017) provided global maps of correction coefficients (substituting the 0.023) and for the original thornthwaite method respective correction coefficients are given in Aschonitis et al. (2022). Both datasets are stored in Pangae database in various resolutions including the ~1 km.

 Eisenhauer J, 2003 (Ref 1)

 Aschonitis V, et al. 2017 (Ref 2)

 Aschonitis V, et al. 2022 (Ref 3)

Are sufficient details of methods and materials provided to allow replication by others?

Yes

Is the rationale for creating the dataset(s) clearly described?

Yes

Are the datasets clearly presented in a useable and accessible format?

Yes

Are the protocols appropriate and is the work technically sound?

Yes

Reviewer Expertise:

Hydrology, Hydroclimatology, Soil Science, Environmental Modelling

I confirm that I have read this submission and believe that I have an appropriate level of expertise to confirm that it is of an acceptable scientific standard, however I have significant reservations, as outlined above.

## References

[1] : Regression through the Origin. *Teaching Statistics* .2003;25(3) : 10.1111/1467-9639.00136 76-80 10.1111/1467-9639.00136

[2] : High-resolution global grids of revised Priestley–Taylor and Hargreaves–Samani coefficients for assessing ASCE-standardized reference crop evapotranspiration and solar radiation. *Earth System Science Data* .2017;9(2) : 10.5194/essd-9-615-2017 615-638 10.5194/essd-9-615-2017

[3] : Correcting Thornthwaite potential evapotranspiration using a global grid of local coefficients to support temperature-based estimations of reference evapotranspiration and aridity indices. *Earth System Science Data* .2022;14(1) : 10.5194/essd-14-163-2022 163-177 10.5194/essd-14-163-2022

# Response

**Published:** 2025-01-29

**Authors:** Robert Zomer

**Affiliations:** IAFES, Foundation Euro-Mediterranean Center for Climate Change, Sassari, Sardinia, Italy

**Response to Reviewer #1** **Vassilis Aschonitis**, Hellenic Agricultural Organization, Soil and Water Resources Institute, Thessaloniki, Greece The authors express their gratitude to the reviewer for his thoughtful and positive comments, and particularly his evaluation that the work is robust.  See below for specific responses to the comments:

1. “The regressions in Fig.9 and 10 are based on y=ax  instead of y=ax+b. The R2 of the two regression lines are different with the first one providing artificially higher R2. The calculation of R2 for a linear regression with intercept forced to 0 is theoretically problematic and should be avoided (Eisenhauer, 2003). Another reason for not using linear regression with 0 intercept in 1:1 plots is that the regression line neglects information associated to the performance of a model in the low values.”

We do understand the point raised by the reviewer. We had also different feedbacks asking regression line with the intercept forced to 0, because of earlier reviews we received on our previous paper using the Penman-Montieth method submitted to Scientific Data.  While setting regression with 0 intercept may provide limited model evaluation at low value, as the reviewer rightly suggest, on the other side it integrates the systematic modelling biases under/overestimate from low to high values range also in the performance evaluation. To balance out, we have set regression lines forced to 0 to provide the more stringent and conservative option comparing observed vs simulated values. Keeping this in consideration, we did not report the r ^2^ for trend lines in Fig. 9 and 10 (actually, there is one, but this is an oversight) as it seems inappropriate and misleading  However, the r ^2^  values reported in the text and tables are from the regression analysis, and not from the forced regression lines.  As per your guidance, we have now redone Figures 9 and 10.  The trend line no longer is forced through zero, and we have added the r ^2^  to all the graphs in Figures 9 and 10.

1. For the PET methods description, you give only the equations of Hargreaves but not the equations of the other four methods used in this study.

We have now added these to the paper.

1. Just for your consideration, there are newer modifications of Hargreaves and Thornthwaite methods based on ASCE Penman-Monteith method. For the original Hargreaves method, the study of Aschonitis et al. ( 2017) provided global maps of correction coefficients (substituting the 0.023) and for the original thornthwaite method respective correction coefficients are given in Aschonitis et al. (2022). Both datasets are stored in Pangae database in various resolutions including the ~1 km.

The global database of local correction coefficients (substituting the 0.023) is indeed an extremely valuable database to close the gap between PM reference methods and simpler temperature-based methods. The correction coefficients mentioned by the reviewer were produced based on partial weighted average of monthly ratios of alternative ET0 methods compared to the reference PM method. It provides a spatial distribution of correction coefficients based on global gridded data of the period 1950-2000. Aschonitis et al. (2017) have shown that there are not serious deviation of such correction coefficients between 1950-2000 and 2000-2020 period, but as we assume this study under climate change, we can not assume that the same climate dependent ET ratios (and thereafter the correction coefficient) held steady at same location in the far future (up to 2060). This would imply at any site not basic changes in the correlation between temperature (used in Hargreaves equations) with others variables (RH, Wind Speed, solar radiation) additionally used in the PM equation, to maintain relative divergence between Hargreaves and Penman-Monteith values steady (and in turn correction coefficients) from historical to future conditions. Valuable effort to improve use of correction coefficient for future climate conditions, could possibly define correction factors that would be climate explicit (possibly in relation to temperature regimes), rather than just spatially explicit.  To acknowledge the value of corrected parameters to improve reliability of Hargreaves equation, we have added the following in technical validation section: *“Among other factors, the significant and specific influence of relative humidity and wind speed lead to particular deviations of results across broad climate regions between Hargreaves and Penman-Monteith model results (Allen et al., 1998). This uncertainty has led to several successful attempts (Tegos et al. 2017,* *Aschonitis et al. 2017) to provide calibration and correction factors map for Hargreaves estimates, based on parametric regression between Hargreaves PET and PM ET0 monthly values for historical condition, that can close the gap between results from Hargreaves and PM methods.  While recognizing the value of such efforts, expansion on the use of these parametric corrections have still been quite limited on future projections, This would require at any site not basic changes in correlation between temperature (used in Hargreaves equations) with the others variables (RH, Wind Speed, solar radiation) additionally used in the PM equation, to maintain relative divergence between Hargreaves and Penman-Monteith values steady (and in turn correction coefficients) from historical to future conditions.”* **References**

1. Allen RG, Pereira LS, Raes D, et al.: Crop evapotranspiration —guidelines for computing crop water requirements. FAO irrigation and drainage paper 56. *Food Agr Org.* Rome,1998. Reference Source
2. Eisenhauer J: Regression through the Origin. *Teaching Statistics. *2003; **25** (3): 76-80 | Publisher Full Text
3. Aschonitis V, Papamichail D, Demertzi K, et al.: High-resolution global grids of revised Priestley–Taylor and Hargreaves–Samani coefficients for assessing ASCE-standardized reference crop evapotranspiration and solar radiation. *Earth System Science Data. *2017; **9** (2): 615-638 | Publisher Full Text
4. Aschonitis V, Touloumidis D, ten Veldhuis M, et al.: Correcting Thornthwaite potential evapotranspiration using a global grid of local coefficients to support temperature-based estimations of reference evapotranspiration and aridity indices. *Earth System Science Data. *2022; **14** (1): 163-177 | Publisher Full Text
5. Tegos A, Malamos N, Efstratiadis A, et al.: Parametric Modelling of Potential Evapotranspiration: A Global Survey. *Water. *2017; **9** (10): | Publisher Full Text
