# Supplementary material for: CMIP6-based global estimates of future aridity index and potential evapotranspiration for 2021-2060
Source: Open Res Eur. 2025 Mar 6;4:157. Originally published 2024 Jul 29. [Version 3] doi: 10.12688/openreseurope.18110.3 (PMC11806256; doi:10.12688/openreseurope.18110.3)
Supplement: Peer review history

# Response

**Published:** 2025-01-29

**Authors:** Robert Zomer

**Affiliations:** IAFES, Foundation Euro-Mediterranean Center for Climate Change, Sassari, Sardinia, Italy

**Reviewer Report - 3** 2 Views

**Approved with reservations**

**27 Sep 2024** VERSION 1

**Aristoteles Tegos**, National Technical University Athens, Athens, Greece  

**Reviewer Comments:**

 The manuscript presents a high-resolution average annual and monthly global estimates of potential evapotranspiration (PET) and aridity index (AI) for 22 CMIP6 Earth System Models for two future (2021-2041; 2041-2060) and two historical (1960-1990; 1970–2000) time periods. The paper is well written but needs several modifications to include recent scientific findings.

 My main concern is the lack of the discussion for the required local calibration of Hargreaves model which has been already  proved to be necessary given the physical complexity of PET phenomenon (Tabari and Talaee 2011( Ref 1), Haslinger and Bartsc 2016  (Ref 2)). The same, using the same global database, has been highlighted by Tegos et al. 2017  (Ref 3)  where the local calibration of temperature-base or simplified extraterrestrial are  irreplaceable for improving the performance of simplified PET approaches (also Tegos et al 2015 (Ref 4)). I would recommend further discussion on that in order to provide limitations of the current dataset and also future improvements especially in geographic where temperature-radiation approaches are failed to provide reliable PET estimates such as Amazonios etc (Tegos et al .2017 (Ref 5)).

**Author Response:**

 We do reckon the added value of parametric corrections for the Hargreaves equations, in order to reduce gap with PM Monteith and especially some specific climate broader areas. The global database of corrected parametric equations for the Hargreaves eq (Tegos et al. 2017) can provide valuable information to improve accuracy of the Hargreaves method in such regions. Tegos et al. (2017) provide a spatial distribution of corrected parametric coefficients, based on regression between Hargreaves PET and PM ET0 monthly values for historical periods (based on historical observation available from CLIMWAT 2.0 database). As mentioned also by the first reviewer, several studies have tested calibration of Hargraves for current climate conditions. While recognizing the value of such efforts, we can not yet assume that such parametric regression (and thereafter the parameter correction) hold steady at same location in the far future (up to 2060). This would imply at any site not basic changes in the correlation between temperature (used in Hargreaves equations) with the others variables (RH, Wind Speed, solar radiation) additionally used in the PM equation, to maintain relative divergence between Hargreaves and Penman-Monteith values steady (and in turn correction coefficients) from historical to future conditions. In fact, none of the mentioned citations (Tabari and Talaee 2011, Haslinger and Bartsc 2016, Tegos et al. 2017, Tegos et al 2015) uses corrected parameters factors to climate projections. Valuable effort to improve use of correction coefficient for future climate conditions, could possibly define correction factors that would be climate explicit (possibly in relation to Temperature regimes), rather than just spatially explicit.  Any shift in the climate regime, due to climate change, would be associated with the shift of corrected parameters. To Acknowledge the value of corrected parameters to improve reliability of Hargreaves equation, we have added the following in technical validation section *Among other factors, the significant and specific influence of relative humidity and wind speed lead to particular deviations of results across broad climate regions between Hargreaves and Penman-Monteith model results (Allen et al., 1998). This uncertainty has led to several successful attempts (Tegos et al. 2017,* *Aschonitis et al. 2017) to provide calibration and correction factors map for Hargreaves estimates, based on parametric regression between Hargreaves PET and PM ET0 monthly values for historical condition, that can close the gap between results from Hargreaves and PM methods.  While recognizing the value of such efforts, expansion on the use of these parametric corrections have still been quite limited on future projections, This would require at any site not basic changes in correlation between temperature (used in Hargreaves equations) with the others variables (RH, Wind Speed, solar radiation) additionally used in the PM equation, to maintain relative divergence between Hargreaves and Penman-Monteith values steady (and in turn correction coefficients) from historical to future conditions.*

**Reviewer Comments:**

*Among the various equations employed for estimating PET.* Suggest to add as citation the paper which presents a detailed review of PET model (Mchmahon et al 2016).

**Author Response:**

 Citation was added

**Reviewer Comments:**

*The Hargreaves (* *Hargreaves, 1994b* *) equation demonstrated the nearest results to the classic PM (* *Droogers & Allen, 2002* *;* *Itenfisu et al., 2003* *). The Hargreaves method with substantially less data requirements, i.e., requiring only measured values of maximum and minimum temperatures and precipitation, was recommended for more general use and therefore is considered more appropriately applicable to data scarce regions and situations (* *Droogers & Allen, 2002* *). For the purposes of this paper, to maintain clarity, we refer to reference evapotranspiration calculated using the Penman-Monteith as “ET _0_” and calculations using the Hargreaves equation as “PET”. *Please refer to my above comment on the historical criticism of the uncalibrated Hargreaves model. The txt must provide more details in the point. 

**Author Response:**

 See point above

**Reviewer Comments:**

 There is a need for additional discussion of Aridity index (Vicente-Serrano et al. 2010 (Ref 6)). The original index was based on the use of the PET Thornthwaite model however recent a research has been criticized the use Thornthwaite model for assessing aridity severity (Tegos et al. 2023 (Ref 7)) and other PET models are recommended.  

**Author Response:**

 Indeed, some of the analyses results in the paper highlighted precisely the higher level of accuracy of Hargreaves respect to Thornthwaite method, among temperature-based methods. Herein, the choice of using Hargreaves rather ThornTwhaite for the calculation was substantially justified. Following this reviewer insight, we find some relevant merit to add in the usage note the following: *Aridity Index provides estimate meteorological droughts on annual temporal scale, while other methods (e.g. SPEI, PSDI) can be further implemented using these and Worldclim datasets to derive other more specific agrometeorological drought indicators at monthly and seasonal scale (Vicente-Serrano et al. 2010; Tegos et al. 2023).*

**References**

 1. Tabari H, Talaee P: Local Calibration of the Hargreaves and Priestley-Taylor Equations for Estimating Reference Evapotranspiration in Arid and Cold Climates of Iran Based on the Penman-Monteith Model. *Journal of Hydrologic Engineering. *2011; **16** (10): 837-845 | Publisher Full Text

 2. Haslinger K, Bartsch A: Creating long term gridded fields of reference evapotranspiration in Alpine terrain based on a re-calibrated Hargreaves method. 2015; | Publisher Full Text

 3. Tegos A, Malamos N, Efstratiadis A, et al.: Parametric Modelling of Potential Evapotranspiration: A Global Survey. *Water. *2017; **9** (10): | Publisher Full Text

 4. Tegos A, Malamos N, Koutsoyiannis D: A parsimonious regional parametric evapotranspiration model based on a simplification of the Penman–Monteith formula. *Journal of Hydrology. *2015; **524** 708-717 | Publisher Full Text

 5. McMahon T, Finlayson B, Peel M: Historical developments of models for estimating evaporation using standard meteorological data. *WIREs Water. *2016; **3** (6): 788-818 | Publisher Full Text

 6. Vicente-Serrano S, Beguería S, López-Moreno J: A Multiscalar Drought Index Sensitive to Global Warming: The Standardized Precipitation Evapotranspiration Index. *Journal of Climate. *2010; **23** (7):1696-1718 | Publisher Full Text

 7. Tegos A, Stefanidis S, Cody J, et al.: On the Sensitivity of Standardized-Precipitation-Evapotranspiration and Aridity Indexes Using Alternative Potential Evapotranspiration Models. *Hydrology. *2023; **10** (3): | Publisher Full Text
